# Three New Norsesquiterpenoids from the Twigs and Leaves of *Nanhaia speciosa*

**DOI:** 10.3390/molecules31214025

**Published:** 2026-11-09

**Authors:** Zhixin Liu, Jie Xu, Yuan Liu, Lei Yang, Danyu Huang, Song Ang, Pan Luo, Dongli Li

**Affiliations:** 1 School of Pharmacy and Food Engineering, Wuyi University, Jiangmen 529020, China; 2 State Key Laboratory of Quality Research in Chinese Medicine, Macau University of Science and Technology, Macau 999078, China

**Keywords:** *Nanhaia speciosa*, terpenoid, norsesquiterpenoid, antiproliferative activity

## Abstract

Phytochemical investigation of the 95% ethanol extract from the twigs and leaves of *Nanhaia speciosa* (Champ. ex Benth.) J. Compton & Schrire led to the isolation of twelve terpenoids. They consist of three new norsesquiterpenoids (**1**–**3**), six known norsesquiterpenoids (**4**–**9**), two known sesquiterpenoids (**10**–**11**), and one known monoterpenoid (**12**). The structures of the previously undescribed compounds were elucidated by analysis of spectroscopic data and quantum chemical calculations. Notably, compound **2** features an oxidative cleavage between C-5 and C-6 of the six-membered ring, yielding a highly rare *seco*-norsesquiterpenoid skeleton. Furthermore, compound **10** exhibited moderate antiproliferative activity against A549 cells (IC_50_ = 13.7 ± 1.8 μM). This study significantly enriches the chemical profile of the aerial parts of *N. speciosa*.

## 1. Introduction

*Nanhaia speciosa* (Champ. ex Benth.) J. Compton & Schrire (*Fabaceae*)*,* a leguminous liana of the genus *Nanhaia* (comprising two species: *Nanhaia fordii* and *N. speciosa*), is predominantly distributed in tropical and subtropical regions of China, including Guangdong, Guangxi, and Hainan provinces. Its roots, commonly used for medicinal purposes, are known as “Niudali”. The medicinal history of Niudali dates back to the late Ming and early Qing dynasties, with records indicating its efficacy in nourishing the lungs, moistening dryness, promoting blood circulation, and relaxing tendons [1]. In modern times, as a medicinal and edible plant, Niudali is often prepared in soups or macerated in wine to release its bioactive constituents, serving to strengthen muscles and bones [2].

Previous phytochemical and pharmacological studies showed that *N. speciosa* contains diverse bioactive constituents, including flavonoids [3,4], alkaloids [5], triterpenoids [6,7], and other compounds [8,9,10], which contribute to various pharmacological effects such as immune-enhancing [11], anticancer [8,12], and anti-inflammatory activities [13]. To date, phytochemical and biological studies on *N. speciosa* have been predominantly directed toward its roots, with eight reported investigations [5,6,7,8,9,14,15,16]. In contrast, the stems have been examined in only three studies [4,10,17]. Notably, no research has been published on the chemical constituents or biological activities of the twigs and leaves. Therefore, the present study aims to fill this gap by investigating the twigs and leaves of *N. speciosa*, thereby providing new insights into the potential bioactive compounds from this previously unexplored plant part.

Moreover, the twigs and leaves of *N. speciosa* constitute the primary by-products generated during the processing of its medicinal roots. Although a small proportion is occasionally utilized as animal feed or for herbal tea preparation, the vast majority are discarded as waste, and their potential pharmacological properties remain largely unexplored and underutilized. The effective utilization of these non-traditional medicinal parts is therefore important for enhancing the comprehensive value of this plant [18]. In fact, the aerial parts of medicinal plants are frequently rich in diverse secondary metabolites with potential bioactivities, making them valuable sources for the discovery of bioactive natural products [19].

**Figure 1:**
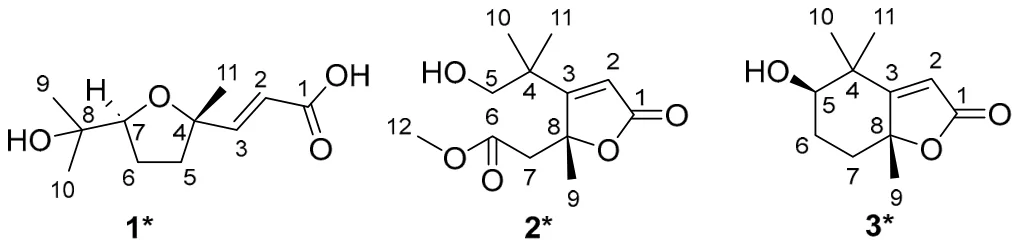
The chemical structures of compounds **1**–**3**.

As part of our ongoing phytochemical investigations of bioactive constituents from traditional medicinal plants [20,21,22,23,24], the aerial parts of *N. speciosa* were selected for systematic chemical study to explore their chemical composition and potential bioactivities. As a result, twelve terpenoids, including three previously undescribed norsesquiterpenoids (**1**–**3**) (Figure 1) and nine known terpenoids (**4**–**12**), were isolated and structurally characterized. All isolated compounds were further evaluated for their antiproliferative activity against A549 cells. Among them, compound **10** showed moderate activity, with an IC_50_ value of 13.7 ± 1.8 μM. This study substantially expands the chemical diversity of *N. speciosa*.

## 2. Results

**Figure 2:**
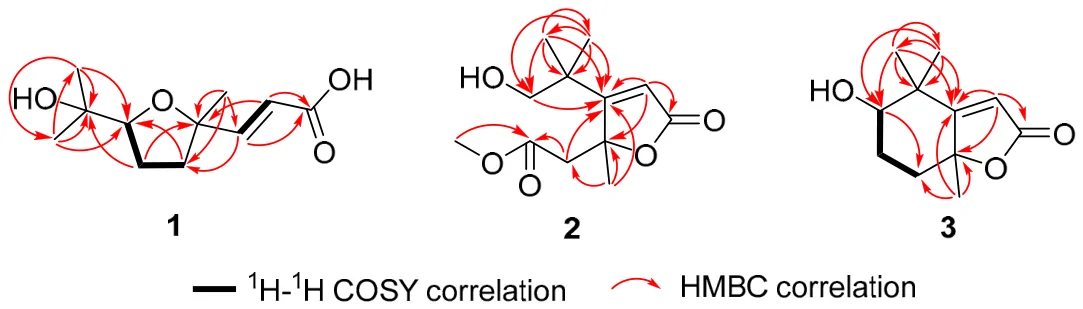
The key ^1^H–^1^H COSY and HMBC correlations of compounds **1**–**3**.

**Figure 3:**
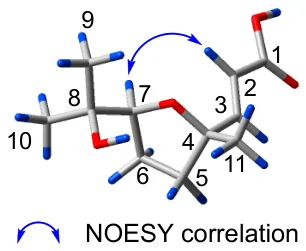
The key NOESY correlations of compound **1**.

**Figure 4:**
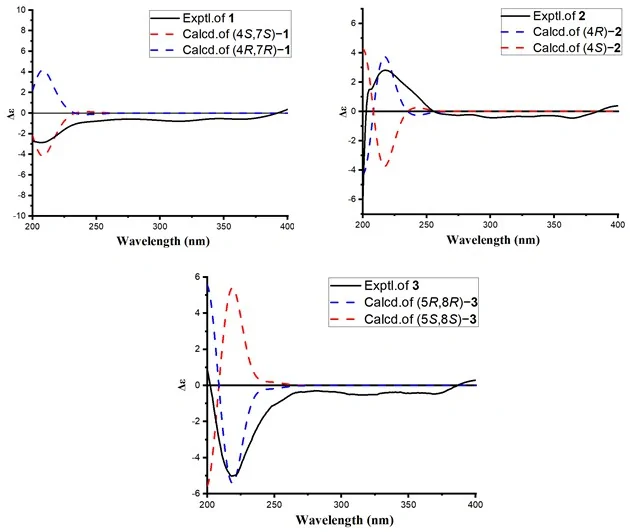
Experimental and calculated ECD spectra of compounds **1**–**3**.

(*E*)-3-((2*S*,5*S*)-5-(2-Hydroxypropan-2-yl)-2-methyltetrahydrofuran-2-yl)acrylic acid (**1**) was obtained as a yellow oil. Its molecular formula (C_11_H_18_O_4_) was determined by analysis of the (+)-HRESIMS peak at *m*/*z* 237.1095 ([M + Na]^+^, calcd. 237.1097), requiring a degree of unsaturation of three. The IR spectrum showcased absorptions for a hydroxy group (3368 cm^−1^) and a carboxyl group (1657 cm^−1^). Eleven carbon signals were observed in the ^13^C NMR spectrum of compound **1** (Table 1). After analysis of the HSQC spectrum, they were assigned as three sp^2^ carbons (*δ*_C_ 173.7, 150.9 and 123.2), two sp^3^ quaternary carbons (*δ*_C_ 83.8 and 72.4), one sp^3^ methine carbon (*δ*_C_ 87.1), two sp^3^ methylene carbons (*δ*_C_ 38.4 and 27.2), and three methyl carbons (*δ*_C_ 26.9, 25.9 and 25.7). The ^1^H NMR spectrum showed signals of three sets of methyl protons [*δ*_H_ 1.33 (3H, s), 1.17 (3H, s) and 1.14 (3H, s)]; one oxygenated methine proton at *δ*_H_ 3.81 (1H, t, *J* = 6.9 Hz); and two olefinic protons at *δ*_H_ 6.71 (1H, d, *J* = 15.5 Hz) and 5.95 (1H, d, *J* = 15.5 Hz). The aforementioned data account for a degree of unsaturation of two, indicating the presence of a monocyclic system. Two spin systems, namely H-2–H-3 and H-5–H-6–H-7, were detected in the ^1^H–^1^H COSY spectrum (Figure 2). The HMBC spectrum showed key correlations from H-9 to C-7/C-8/C-10, H-10 to C-7/C-8/C-9, H-11 to C-3/C-4/C-5, H-6 to C-4, H-5 to C-7, H-3 to C-1/C-2/C-5, and H-2 to C-1/C-3 (Figure 2). The planar structure of compound **1** was thus established, with a core skeleton identical to that of (2R*,5R*,1′*E*,3′*E*)-5-(1″-hydroxy-1″-methylethyl)-2-methyl-2-(4′-methylhexa-1′,3′,5′-trienyl)tetrahydrofuran [25]. In contrast to the known compound, the polyene chain at C-4 in this case may undergo oxidative cleavage followed by oxidation to the carboxylic acid, yielding compound **1**. The key NOESY between H-7 and H-2 suggested that H-7 and H-2 are co-facial, and these were assigned as α-oriented, with CH_3_-11 having a β-orientation (Figure 3). Finally, the absolute configuration of compound **1** was unambiguously determined as 4*S*,7*S* by comparison of calculated and experimental electronic circular dichroism (ECD) spectra (Figure 4).

Methyl (*R*)-2-(3-(1-hydroxy-2-methylpropan-2-yl)-2-methyl-5-oxo-2,5-dihydrofuran-2-yl)acetate (**2**) was obtained as a colourless oil. Its molecular formula (C_12_H_18_O_5_) was determined by analysis of the (+)-HRESIMS peak at *m*/*z* 265.1039 ([M + Na]^+^, calcd. 265.1046), indicating a degree of unsaturation of four. The IR spectrum showed absorption for a carbonyl group (1747 cm^−1^). Twelve carbon signals were observed in the ^13^C NMR spectrum of compound **2** (Table 1). After analysis of the HSQC spectrum, they were assigned as four sp^2^ carbons (*δ*_C_ 177.8, 171.0, 170.2 and 119.7), two sp^3^ quaternary carbons (*δ*_C_ 87.4 and 40.6), two sp^3^ methylene carbons (*δ*_C_ 71.1 and 42.4), and four methyl carbons (*δ*_C_ 52.5, 26.9, 26.7 and 23.9). The ^1^H NMR spectrum showed signals of four sets of methyl protons [*δ*_H_ 3.66 (3H, s), 1.65 (3H, s), 1.29 (3H, s) and 1.26 (3H, s)], which include an oxygenated methyl proton at *δ*_H_ 3.66 (3H, s); one set of oxygenated methylene proton at *δ*_H_ 3.54 (1H, d, *J* = 11.3 Hz) and 3.50 (1H, d, *J* = 10.3 Hz); and one olefinic proton at *δ*_H_ 5.97 (1H, s). The aforementioned data account for a degree of unsaturation of three, suggesting a monocyclic carbon skeleton of **2**. The HMBC spectrum showed key correlations from H-9 to C-3/C-7/C-8, H-10 to C-3/C-4/C-5/C-11, H-11 to C-3/C-4/C-5/C-10, H-2 to C-1/C-3/C-8, and H-7/H-12 to C-6 (Figure 2). Thus, the planar structure of compound **2** was elucidated. The absolute configuration of **2** was ultimately determined as 8*R* through comparison of calculated and experimental ECD spectra (Figure 4). Biosynthetically, compound **2** shares a common norsesquiterpenoid origin with compounds **3**–**6**. However, it features an oxidative cleavage between C-5 and C-6 of the six-membered ring, yielding a distinct *seco*-norsesquiterpenoid skeleton. Such a ring-cleaved architecture is highly rare within this structural class. To the best of our knowledge, compound **2** represents the first naturally occurring norsesquiterpenoid of this type possessing the cleaved six-membered ring skeleton.

(5*R*,7a*R*)-5-Hydroxy-4,4,7a-trimethyl-5,6,7,7a-tetrahydrobenzofuran-2(4H)-one (**3**) was isolated as a colourless oil. Its molecular formula (C_11_H_16_O_3_) was determined by analysis of the (+)-HRESIMS peak at *m*/*z* 219.0989 ([M + Na]^+^, calcd. 219.0991), requiring a degree of unsaturation of four. The IR spectrum showcased absorptions for a hydroxy group (3423 cm^−1^) and a carbonyl group (1730 cm^−1^). Eleven carbon signals were observed in the ^13^C NMR spectrum of compound **3** (Table 1). After analysis of the HSQC spectrum, they were assigned as three sp^2^ carbons (*δ*_C_ 180.8, 172.0 and 114.5), two sp^3^ quaternary carbons (*δ*_C_ 86.6 and 41.5), one sp^3^ methine carbon (*δ*_C_ 77.9), two sp^3^ methylene carbons (*δ*_C_ 35.8 and 28.0), and three methyl carbons (*δ*_C_ 25.9, 24.6 and 18.1). The ^1^H NMR spectrum showed signals of three sets of methyl protons [*δ*_H_ 1.57 (3H, s), 1.33 (3H, br s) and 1.17 (3H, br s)]; one oxygenated methine proton at *δ*_H_ 3.37 (1H, t, *J* = 5.6 Hz); and one olefinic proton at *δ*_H_ 5.73 (1H, s). One spin system, namely H-5–H-6–H-7, was detected in the ^1^H–^1^H COSY spectrum (Figure 2). The HMBC spectrum showed key correlations from H_3_-9 to C-3/C-7/C-8, H_3_-10 to C-3/C-4/C-5/C-11, H_3_-11 to C-3/C-4/C-5/C-10, H-2 to C-1/C-3/C-8, and H-5 to C-7 (Figure 2). Based on the above analyses, compound **3** was found to possess the same planar structure as the previously reported 3-hydroxy-2,2,6-trimethyl-7-oxabicyclo[4.3.0]non-9-en-8-one [26]. However, notable discrepancies in several ^13^C NMR chemical shifts suggested that they may be C-5 or C-8 epimers. The NOESY experiment was conducted to determine the relative configuration of compound **3**. Unfortunately, no significant NOESY cross-peaks were observed. Therefore, quantum chemical calculations of NMR chemical shifts were performed. By comparing the experimental ^13^C NMR data of compound **3** with the calculated values of two putative stereoisomers (Figure S48), the relative configuration of compound **3** was determined to be 5S*,8S*. Finally, comparison of the experimental and calculated ECD spectra confirmed the absolute configuration of **3** as 5*R*,8*R* (Figure 4), establishing that this compound is the enantiomer of pedatisectaene A [27].

The known compounds were identified as loliolide (**4**) [28], (−)-10-hydroxydihydroactinidiolide (**5**) [29], dehydrololiolide (**6**) [30], (+)-dehydrovomifoliol (**7**) [31], annuionone B (**8**) [32], robinlin (**9**) [33], (+)-1-methyl-abscisic-6-acid (**10**) [34], 2*E*,4*Z*-abscisic acid (**11**) [35], and paeoveitol B (**12**) [36] after comparison with reported NMR spectroscopic data. All these terpenoids are reported from the *Nanhaia* genus for the first time.

The antiproliferative activities of all isolated compounds against A549 cells were evaluated using the 3-(4,5-dimethylthiazol-2-yl)-2,5-diphenyltetrazolium bromide (MTT) assay (Figure S49). Compounds were first screened at 20 µM. Only compound **10** was worth investigating based on cell viability, while the others were inactive. As shown in Figure S50, compound **10** displayed moderate antiproliferative activity against A549 cells, with an IC_50_ value of 13.7 ± 1.8 μM.

## 3. Discussion

To date, phytochemical investigations of *N. speciosa* have mainly focused on its stems and roots, with no reports on the chemical constituents of the aerial parts (twigs and leaves) [5,6,7,8,9,14,15,16]. Therefore, this study represents the first systematic chemical investigation of the twigs and leaves of *N. speciosa*, thereby addressing this gap in its phytochemical profile. The results showed that terpenoids **1**–**12** isolated in this study were all obtained from this genus for the first time, greatly enriching the chemical diversity of *N. speciosa*. Notably, compounds **1**–**3** are new compounds, and compound **2** features an oxidative cleavage between C-5 and C-6 of the six-membered ring, yielding a highly rare *seco*-norsesquiterpenoid skeleton. Furthermore, the isolated compounds were evaluated for their antiproliferative activity against A549 cells, and only compound **10** exhibited moderate bioactivity with an IC_50_ value of 13.7 ± 1.8 μM. However, the specific mechanism underlying the antiproliferative activity of this compound remains unclear and warrants further in-depth investigation. In addition, the compounds isolated in this study, particularly the new compounds, were only preliminarily evaluated for antiproliferative activity against A549 cells, and screening for other activities such as cytotoxicity against other cancer cell lines, anti-inflammatory, antioxidant, and antibacterial activities will also lay the foundation for the high-value utilization of the twigs and leaves of *N. speciosa*.

## 4. Materials and Methods

### 4.1. General Experimental Procedures

^1^H NMR and ^13^C NMR data were recorded on a Bruker AVANCE NEO 500 or 600 spectrometer (Bremen, Germany) with methanol-*d*_4_, acetone-*d*_6_ and chloroform-*d* as solvents. Chemical shift values were expressed in *δ* (ppm) relative to tetramethylsilane (TMS) as the internal standard. The HR-ESI-MS was performed using an ESI ion source Q-Exactive mass spectrometer (Thermo Fisher Scientific, Waltham, MA, USA). Infrared spectroscopy (IR) spectra were recorded with KBr pellets on a Fourier Transform NICOLET iS5 Infrared Spectrometer (Thermo Fisher Scientific, Waltham, MA, USA). The optical rotations were recorded in acetone at 25 °C on an Anton Paar MCP 200 automatic polarimeter (Graz, Austria). Circular dichroism (CD) spectra were collected at room temperature on a Chirascan spectrometer (Leatherhead, UK) using a standard cell with a 0.2 cm path length. Column chromatographic separations were conducted on stationary phases of silica gel (100–200 and 200–300 mesh, Qingdao Marine Chemical Factory, Qingdao, China), C_18_ silica gel (Fu Silysia Chemical Ltd., Kasugai, Japan; 100–20/45 mesh), Sephadex LH-20 (Pharmacia Biotech, Piscataway, NJ, USA), and MCI gel (CHP-20P, Mitsubishi Chemical Industries Co., Ltd., Tokyo, Japan). Semi-preparative HPLC was conducted on an Agilent 1500-series system (Agilent, Santa Clara, CA, USA) equipped with a 2489 DAD detector, utilizing a SunFire^®^ C_18_ OBD column (Waters, Milford, MA, USA; 250 mm × 10 mm). Solvents for column chromatography and HPLC were of analytical grade (Guangzhou Chemical Reagents Company Ltd., Guangzhou, China) and chromatographic grade (Thermo Fisher Scientific, Waltham, MA, USA), respectively. All solvent ratios given are volume/volume (*v*/*v*) unless otherwise stated.

### 4.2. Plant Material

The twigs and leaves of *N. speciosa* were collected in July 2024 in Yunfu City, Guangdong Province, People’s Republic of China. The plant was identified by Senior Engineer Feiyan Zeng of the South China Botanical Garden, Chinese Academy of Sciences. A voucher specimen (202407NS) was preserved in the School of Pharmacy and Food Engineering, Wuyi University, Jiangmen, China.

### 4.3. Extraction and Isolation

Dried aerial parts (35 kg) of *N. speciosa* were powdered and macerated with 95% ethanol at room temperature (5 × 25 L, 7 days each). The combined extracts were concentrated under reduced pressure to afford the crude ethanol extract (645 g). The extract was suspended in 4.0 L of distilled water and then sequentially partitioned with petroleum ether (PE), ethyl acetate (EtOAc), and *n*-butanol (*n*-BuOH) to yield the PE-soluble fraction (343 g), EtOAc-soluble fraction (54.6 g), and *n*-BuOH-soluble fraction (51.1 g), respectively.

The EtOAc-soluble fraction (54.6 g) was split into six fractions (EA1–EA6) using an MCI gel column (MeOH/H_2_O, 10:90–100:0). EA2 (10.5 g) was separated into nine fractions (EA2B1–EA2B9) by using silica gel CC (PE/EtOAc, 20:1–1:4). EA2B8 (1.2 g) was separated into six fractions (EA2B8C1–EA2B8C6) by using silica gel CC (CH_2_Cl_2_/MeOH, 200:1–1:4). EA2B8C1 was purified by semi-preparative HPLC (MeOH/H_2_O, 27/73, 3.0 mL/min) to yield compound **3** (1.1 mg, *t*_R_ = 35.2 min) and compound **4** (9.7 mg, *t*_R_ = 52.6 min). EA2B8C4 was purified by semi-preparative HPLC (MeOH/H_2_O, 35/65, 3.0 mL/min) to yield compound **1** (2.3 mg, *t*_R_ = 36.7 min) and compound **12** (9.8 mg, *t*_R_ = 47.3 min). EA2B6 was purified by semi-preparative HPLC (MeOH/H_2_O, 25/75, 3.0 mL/min) to yield compound **6** (3.4 mg, *t*_R_ = 35.2 min). EA2B9 (4.9 g) was separated into six fractions (EA2B9C1–EA2B9C6) by using silica gel CC (CH_2_Cl_2_/MeOH, 100:1–1:4). EA2B9C1 (400.0 mg) was further separated using Sephadex LH-20 (MeOH) to obtain two fractions (EA2B9C1D1–EA2B9C1D2). EA2B9C1D1 was purified by semi-preparative HPLC (MeOH/H_2_O, 30/70, 3.0 mL/min) to yield compound **8** (3.5 mg, *t*_R_ = 11.8 min), compound **7** (5.2 mg, *t*_R_ = 14.5 min), compound **2** (4.8 mg, *t*_R_ = 18.2 min), and compound **5** (1.5 mg, *t*_R_ = 28.3 min). EA3 (11.0 g) was separated into six fractions (EA3B1–EA3B6) by using silica gel CC (PE/EtOAc, 200:1–1:4). EA3B2 (3.1 g) was split into five fractions (EA3B2C1–EA3B2C5) using an ODS column (MeOH/H_2_O, 50:50–100:0, *v*/*v*). EA3B2C1 was purified by semi-preparative HPLC (MeOH/H_2_O, 32/68, 3.0 mL/min) to yield compound **10** (5.2 mg, *t*_R_ = 52.3 min). EA3B2C3 was purified by semi-preparative HPLC (MeOH/H_2_O, 43/57, 3.0 mL/min) to yield compound **11** (4.9 mg, *t*_R_ = 18.3 min). EA3B4 (2.1 g) was separated into four fractions (EA3B4C1–EA3B4C4) by using silica gel CC (PE/EtOAc, 80:1–1:1). EA3B4C1 was purified by semi-preparative HPLC (MeOH/H_2_O, 45/55, 3.0 mL/min) to yield compound **9** (2.8 mg, *t*_R_ = 29.5 min).

### 4.4. Compound Characterization

(*E*)-3-((2*S*,5*S*)-5-(2-Hydroxypropan-2-yl)-2-methyltetrahydrofuran-2-yl)acrylic acid (**1**): yellowish oil; [*α*${]}_{D}^{25}$ −80.4 (*c* 1.15, acetone); UV (CH_3_CN) λ_max_ (log *ε*): 209 (0.61) nm; IR (KBr) ν_max_: 3537, 3368, 1657, 1641, 1630, 1383 cm^−1^; ECD (CH_3_CN) λ_max_ (Δ*ε*) 208 (−2.83); (+)-HRESIMS: *m*/*z* 237.1095 [M + Na]^+^ (calcd. for C_11_H_18_O_4_Na, 237.1097); for NMR data, see Table 1.

Methyl (*R*)-2-(3-(1-hydroxy-2-methylpropan-2-yl)-2-methyl-5-oxo-2,5-dihydrofuran-2-yl)acetate (**2**): colourless oil; [*α*${]}_{D}^{25}$ +73.3 (*c* 2.40, acetone); UV (CH_3_CN) λ_max_ (log *ε*): 216 (0.97) nm; IR (KBr) ν_max_: 2919, 1747, 1732, 1697, 1362 cm^−1^; ECD (CH_3_CN) λ_max_ (Δ*ε*) 217 (2.81); (+)-HRESIMS: *m*/*z* 265.1039 [M + Na]^+^ (calcd. for C_12_H_18_O_5_Na, 265.1046); for NMR data, see Table 1.

(5*R*,7a*R*)-5-Hydroxy-4,4,7a-trimethyl-5,6,7,7a-tetrahydrobenzofuran-2(4H)-one (**3**): colourless oil; [*α*${]}_{D}^{25}$ −43.3 (*c* 0.55, acetone); UV (CH_3_CN) λ_max_ (log *ε*): 215 (0.77) nm; IR (KBr) ν_max_: 3423, 2922, 1730, 1263 cm^−1^; ECD (CH_3_CN) *λ*_max_ (Δ*ε*) 219 (−5.01); (+)-HRESIMS: *m*/*z* 219.0989 [M + Na]^+^ (calcd. for C_11_H_16_O_3_Na, 219.0991); for NMR data, see Table 1.

### 4.5. Quantum Chemical Calculation of ^13^C NMR Data of 3

The relative configuration of compound **3** was determined by analysis of calculated ^13^C NMR. The structures of **3** with putative configurations (5R*,8R* and 5R*,8S*) were constructed using ChemDraw 20.0.0.41, converted to 3D models, and subsequently subjected to a systematic conformational search in Spartan’14. All conformers within 4 kcal/mol of the global minimum were retained. These conformers were then subjected to gas-phase geometry optimization at the B3LYP/6-31G(d) level of theory using Gaussian 09. Subsequently, NMR shielding tensors were calculated with the GIAO (gauge-independent atomic orbital) method at the mPW1PW91/6-311G(d,p) level, employing the polarizable continuum model (PCM) for chloroform as the solvent. The calculated ^1^H and ^13^C NMR chemical shifts were compared with the experimental data using the correlation coefficient (R^2^), mean absolute error (MAE), and DP4^+^ probability analysis. As summarized in Table S4, the 5*R*,8*R* configuration exhibited the best agreement with the experimental NMR data, thereby allowing the unambiguous assignment of the relative configuration of **3** as 5R*,8R*.

### 4.6. Quantum Chemical ECD Calculations of ***1***–***3***

The theoretical calculations of **1**–**3** were performed using Gaussian 09. The possible conformations were initially obtained from the programme Spartan’14 and then optimized at B3LYP/6-31G(d) level in the gas phase. Room-temperature equilibrium populations were calculated according to the Boltzmann distribution law. The ECD calculations were performed using Time Dependent Density Functional Theory (TDDFT) at wB97xd/6-311G(d,p) level in acetonitrile with PCM. The calculated ECD spectra were obtained by weighing the Boltzmann distribution rate of each geometric conformation, and the sigma/gamma value for processing the calculated ECD was 0.3 eV [37].

### 4.7. Reagents

Dulbecco’s modified Eagle’s medium (DMEM) and penicillin–streptomycin (P/S) were purchased from Gibco, and fetal bovine serum (FBS) was obtained from Procell. Cisplatin (CDDP) was purchased from MedChemExpress (MCE). MTT was obtained from TaoSu Biotech Co., Ltd. (Shanghai, China), and dimethyl sulfoxide (DMSO) was purchased from MP Biomedicals. All other reagents were of analytical grade unless otherwise stated.

### 4.8. Antiproliferative Activity Assay

The A549 cell line was cultured in DMEM supplemented with 10% FBS and 1% P/S at 37 °C in a 5% CO_2_ incubator. Cells in the logarithmic growth phase were harvested and seeded into 96-well plates at a density of 5 × 10^3^ cells per well. After 24 h of incubation, the spent medium was replaced with fresh medium containing the test compounds at 20 μM for the initial screen. For the dose–response assay of compound **10**, cells were treated at 10, 20, 40, 60, and 80 μM. Cisplatin (20 μM) was used as a positive control and treated under identical conditions; it reduced A549 viability to 14.1%, validating the assay system. Each concentration was tested in triplicate wells. Cells were then cultured for another 72 h. Subsequently, 10 μL of MTT solution (0.5 mg/mL) was added to each well, and the plates were incubated for 4 h. The supernatant was gently discarded, and 100 μL of DMSO was added to each well. The plates were then shaken for 15 min to fully dissolve the formazan crystals. The absorbance was measured at 490 nm. Dose–response curves were fitted using GraphPad Prism 9.0 with a four-parameter logistic (variable-slope) model, and IC_50_ values were obtained directly from the fitted curves by nonlinear regression analysis. Data were obtained from at least three independent experiments. IC_50_ values from independent experiments were averaged and are reported as mean ± SD.

### 4.9. Statistical Analysis

All experimental results are presented as the mean ± SD from at least three independent experiments, unless otherwise specified. For cell-viability assays, each concentration was tested in triplicate wells, and at least three independent experiments were performed. IC_50_ values were fitted separately for each independent experiment using GraphPad Prism 9.0 with a four-parameter logistic (variable-slope) model, and the resulting values are presented as mean ± SD. Statistical analysis was conducted using GraphPad Prism 9.0. Differences were considered statistically significant at *p* < 0.05. Statistical significance was indicated as * *p* < 0.05, ** *p* < 0.01, *** *p* < 0.001, and **** *p* < 0.0001 compared with the control group.

## 5. Conclusions

In summary, a systematic phytochemical investigation of the aerial parts of *N. speciosa* resulted in the isolation and identification of twelve terpenoids, comprising three previously undescribed norsesquiterpenoids (**1**–**3**) and nine known terpenoids (**4**–**12**). These findings significantly enrich the chemical profile of *N. speciosa*, particularly regarding the terpenoid constituents in its non-traditional medicinal parts. Notably, all these terpenoids are reported from the *Nanhaia* genus for the first time. The compounds were tested against A549 cells. While compound **10** displayed moderate activity, the others were inactive.

## Figures and Tables

**Table 1:** ^1^H (600 MHz) and ^13^C NMR (150 MHz) spectral data of compounds **1**–**3**.

NO	Compound 1		Compound 2		Compound 3	
	*δ*_C_^a^ (ppm)	*δ*_H_^a,c^ (ppm, *J* in Hz)	*δ*_C_^b^ (ppm)	*δ*_H_^b,c^ (ppm, *J* in Hz)	*δ*_C_^b^ (ppm)	*δ*_H_^b,c^ (ppm, *J* in Hz)
1	173.7, C		171.0, C		172.0, C	
2	123.2, CH	5.95, d (15.5)	119.7, CH	5.97, s	114.5, CH	5.73, s
3	150.9, CH	6.71, d (15.5)	177.8, C		180.8, C	
4	83.8, C		40.6, C		41.5, C	
5	38.4, CH_2_	a: 1.92	71.1, CH_2_	a: 3.54, d (11.3)	77.9, CH	3.37, t (5.6)
		b: 1.77		b: 3.50, d (11.3)		
6	27.2, CH_2_	1.86	170.2, C		28.0, CH_2_	a: 1.93, d (13.5)
						b: 1.77–1.69
7	87.1, CH	3.81, t (6.9)	42.4, CH_2_	a: 3.05, d (15.7)	35.8, CH_2_	a: 2.19, d (12.5)
				b: 3.01, d (15.7)		b: 1.50, t (13.6)
8	72.4, C		87.4, C		86.6, C	
9	25.7, CH_3_	1.14, s	26.7, CH_3_	1.65, s	24.6, CH_3_	1.57, s
10	25.9, CH_3_	1.17, s	26.9, CH_3_	1.29, s	25.9, CH_3_	1.33, br s
11	26.9, CH_3_	1.33, s	23.9, CH_3_	1.26, s	18.1, CH_3_	1.17, br s
12			52.5, CH_3_	3.66, s		

^a^ Measured in CD_3_OD. ^b^ Measured in CDCl_3_. ^c^ The indiscernible signals from overlap or the complex multiplicity are reported without designating multiplicity.

## Data Availability

The data supporting the reported results are contained within the manuscript and Supplementary Information.

## Supplementary Materials

The following supporting information can be downloaded at: https://www.mdpi.com/article/10.3390/molecules31214025/s1, Figure S1: Chemical structures of compounds **1**–**12**; Figure S2: ^1^H NMR (600 MHz) spectrum of compound **1** in CD_3_OD; Figure S3: ^13^C NMR (150 MHz) spectrum of compound **1** in CD_3_OD; Figure S4: HSQC spectrum of compound **1** in CD_3_OD; Figure S5: HMBC spectrum of compound **1** in CD_3_OD; Figure S6: ^1^H–^1^H COSY spectrum of compound **1** in CD_3_OD; Figure S7: NOESY spectrum of compound **1** in CD_3_OD; Figure S8: (+)-HRESIMS spectrum of compound **1**; Figure S9: IR (KBr) spectrum of compound **1**; Figure S10: UV spectrum of compound **1**; Figure S11: ^1^H NMR (600 MHz) spectrum of compound **2** in CDCl_3_; Figure S12: ^13^C NMR (150 MHz) spectrum of compound **2** in CDCl_3_; Figure S13: HSQC spectrum of compound **2** in CDCl_3_; Figure S14: HMBC spectrum of compound **2** in CDCl_3_; Figure S15: ^1^H–^1^H COSY spectrum of compound **2** in CDCl_3_; Figure S16: (+)-HRESIMS spectrum of compound **2**; Figure S17: IR (KBr) spectrum of compound **2**; Figure S18: UV spectrum of compound **2**; Figure S19: ^1^H NMR (600 MHz) spectrum of compound **3** in CDCl_3_; Figure S20: ^13^C NMR (150 MHz) spectrum of compound **3** in CDCl_3_; Figure S21: HSQC spectrum of compound **3** in CDCl_3_; Figure S22: HMBC spectrum of compound **3** in CDCl_3_; Figure S23: ^1^H–^1^H COSY spectrum of compound **3** in CDCl_3_; Figure S24: NOESY spectrum of compound **3** in CDCl_3_; Figure S25: (+)-HRESIMS spectrum of compound **3**; Figure S26: IR (KBr) spectrum of compound **3**; Figure S27: UV spectrum of compound **3**; Figures S28–S45: ^1^H NMR and ^13^C NMR spectrum of compounds **4**–**12**; Figure S46: Optimized geometries of 5 dominant conformers of **1** at the B3LYP/6-31G(d) level of theory in acetonitrile (IEFPCM); Figure S47: Optimized geometries of 5 dominant conformers of **2** at the B3LYP/6-31G(d) level of theory in acetonitrile (IEFPCM); Figure S48: Stable conformers used in ^13^C NMR calculation of **3**; Figure S49: Effects of isolated compounds on the viability of A549 cells. Figure S50: Concentration-dependent antiproliferative effect of compound **10** on A549 cells. Tables S1–S3: Important thermodynamic parameters and Boltzmann distributions of the optimized conformation of **1**–**3** (Conf1–3) at B3LYP/6-31G(d) level in gas phase; Table S4: R square (R^2^) of the linear correlations, mean absolute error (MAE) values, and DP4^+^ probability analysis.
