# Supplementary material for: Three New Norsesquiterpenoids from the Twigs and Leaves of Nanhaia speciosa
Source: Molecules. 2026 Nov 9;31(21):4025. doi: 10.3390/molecules31214025 (PMC13764921; doi:10.3390/molecules31214025)
Supplement: Supplementary file 1 [file molecules-31-04025-s001.zip › molecules-4650839-supplementary.pdf]

Supplementary Material

Three New Norsesquiterpenoids from the Twigs and Leaves of *Nanhaia speciosa*

Zhixin Liu [1], Jie Xu [1], Yuan Liu [1], Lei Yang [1], Danyu Huang [2], Song Ang [1], Pan Luo [1,*] and Dongli Li [1,*]

[1] *School of Pharmacy and Food Engineering, Wuyi University, Jiangmen 529020, China*

[2] *State Key Laboratory of Quality Research in Chinese Medicine, Macau University of Science and Technology, Macau 999078, China*

* *Correspondence: luop13@wyu.edu.cn (P.L.); wyuchemldl@126.com (D.L.)*

Abstract: Phytochemical investigation of the 95% ethanol extract from the twigs and leaves of *Nanhaia speciosa* (Champ. ex Benth.) J. Compton & Schrire — led to the isolation of twelve terpenoids. They consist of three new norsesquiterpenoids (**1–3**), six known norsesquiterpenoids (**4–9**), two known sesquiterpenoids (**10–11**), and one known monoterpenoid (**12**). The structures of the previously undescribed compounds were elucidated by analysis of spectroscopic data and quantum chemical calculations. Notably, compound **2** features an oxidative cleavage between C-5 and C-6 of the six-membered ring, yielding a highly rare *seco*-norsesquiterpenoid skeleton. Furthermore, compound **10** exhibited moderate antiproliferative activity against A549 cells (IC_{50} = 13.7 ± 1.8 μM). This study significantly enriches the chemical profile of the aerial parts of *N. speciosa*.

Keywords: *Nanhaia speciosa*, terpenoid, norsesquiterpenoid, antiproliferative activity

Contents

Figure S1. Chemical structures of compounds **1–12**..4

Tables S1–S3. Important thermodynamic parameters and Boltzmann distributions of the optimized conformation of **1–3** (Conf1–3) at B3LYP/6-31G(d) level in gas phase..4

Table S4. R square (R^2) of the linear correlations, mean absolute error (MAE) values, and $DP4^+$ probability analysis..5

Figure S2. ^1H NMR (600 MHz) spectrum of compound **1** in CD_3OD..6

Figure S3. ^{13}C NMR (150 MHz) spectrum of compound **1** in CD_3OD..6

Figure S4. HSQC spectrum of compound **1** in CD_3OD..7

Figure S5. HMBC spectrum of compound **1** in CD_3OD..7

Figure S6. ^1H–^1H COSY spectrum of compound **1** in CD_3OD..8

Figure S7. NOESY spectrum of compound **1** in CD_3OD..8

Figure S8. (+)-HRESIMS spectrum of compound **1** 9

Figure S9. IR (KBr) spectrum of compound **1** 9

Figure S10. UV spectrum of compound **1**..9

Figure S11. ^1H NMR (600 MHz) spectrum of compound **2** in $CDCl_3$..10

Figure S12. ^{13}C NMR (150 MHz) spectrum of compound **2** in $CDCl_3$..11

Figure S13. HSQC spectrum of compound **2** in $CDCl_3$..11

Figure S14. HMBC spectrum of compound **2** in $CDCl_3$..12

Figure S15. ^1H–^1H COSY spectrum of compound **2** in $CDCl_3$..12

Figure S16. (+)-HRESIMS spectrum of compound **2** 13

Figure S17. IR (KBr) spectrum of compound **2**..13

Figure S18. UV spectrum of compound **2**..14

Figure S19. ^1H NMR (600 MHz) spectrum of compound **3** in $CDCl_3$..14

Figure S20. ^{13}C NMR (150 MHz) spectrum of compound **3** in $CDCl_3$..15

Figure S21. HSQC spectrum of compound **3** in $CDCl_3$..15

Figure S22. HMBC spectrum of compound **3** in $CDCl_3$..16

Figure S23. ^1H–^1H COSY spectrum of compound **3** in $CDCl_3$..16

Figure S24. NOESY spectrum of compound **3** in $CDCl_3$..17

Figure S25. (+)-HRESIMS spectrum of compound **3** 17

Figure S26. IR (KBr) spectrum of compound **3**...18

Figure S27. UV spectrum of compound **3**...18

Figures S28–S45. ^{1}H NMR and ^{13}C NMR spectra of compounds **4–12**..19

Figure S46. Optimized geometries of 5 dominant conformers of **1** at the B3LYP/6-31G(d) level of theory in acetonitrile (IEFPCM)...28

Figure S47. Optimized geometries of 5 dominant conformers of **2** at the B3LYP/6-31G(d) level of theory in acetonitrile (IEFPCM)...28

Figure S48. Stable conformers used in ^{13}C NMR calculation of **3**...29

Figure S49. Effects of isolated compounds on the viability of A549 cells.....................................29

Figure S50. Concentration-dependent antiproliferative effect of compound **10** on A549 cells...30

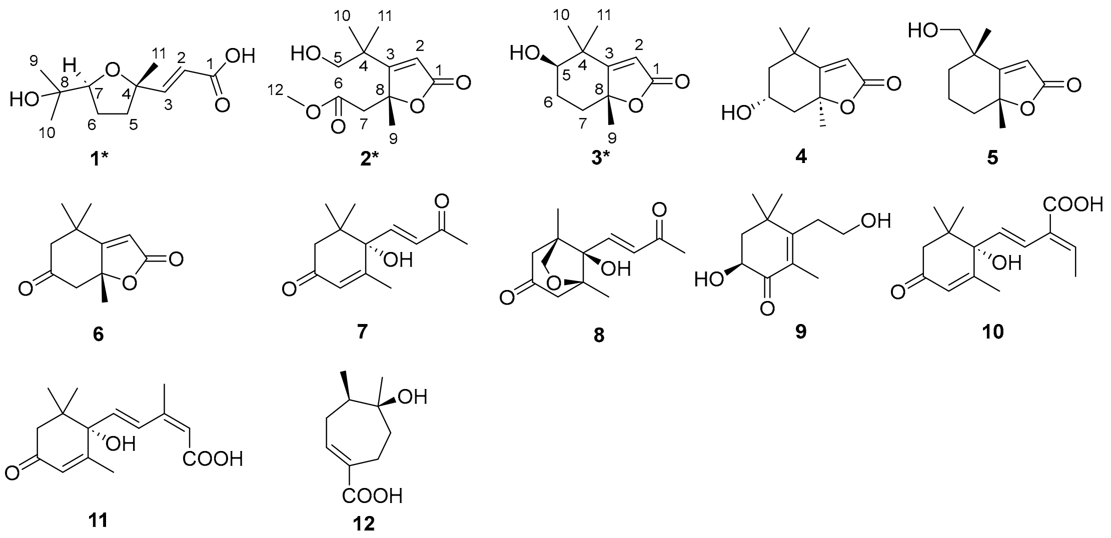

Figure S1. Chemical structures of compounds **1–12**

Table S1. Important thermodynamic parameters and Boltzmann distributions of the optimized conformation of **1** (**Conf1**) at B3LYP/6-31G(d) level in gas phase.

Conformations	Energy (a.u)	Δ G (kcal/mol)	%
**Conf1-1**	−730.7214	0.00	52.4%
**Conf1-2**	−730.7204	0.58	19.7%
**Conf1-3**	−730.7204	0.64	18.0%
**Conf1-4**	−730.7192	1.35	5.4%
**Conf1-5**	−730.7191	1.43	4.7%

Table S2. Important thermodynamic parameters and Boltzmann distributions of the optimized conformation of **2** (**Conf2**) at B3LYP/6-31G(d) level in gas phase.

Conformations	Energy (a.u)	Δ G(kcal/mol)	%
**Conf2-1**	−844.0606	0.00	70.8%
**Conf2-2**	−844.0597	0.56	27.6%
**Conf2-3**	−844.0568	2.40	1.2%
**Conf2-4**	−844.0556	3.13	0.4%
**Conf2-5**	−844.0536	4.37	0.0%

Table S3. Important thermodynamic parameters and Boltzmann distributions of the optimized conformation of **3** (**Conf3**) at B3LYP/6-31G(d) level in gas phase.

Conformations	Energy (a.u)	Δ G(kcal/mol)	%
**Conf3-1**	−654.3190	0.00	35.8%
**Conf3-2**	−654.3189	0.03	33.8%
**Conf3-3**	−654.3188	0.10	30.2%
**Conf3-4**	−654.3134	3.47	0.1%
**Conf3-5**	−654.3130	3.75	0.1%

Table S4. R square (R^2) of the linear correlations, mean absolute error (MAE) values, and DP4$^+$ probability analysis.

NO.	**3** (Experimental)	(5*R**,8*R**)-**3** (Calculated)	AE	(5*R**,8*S**)-**3** (Calculated)	AE
C-1	77.9	78.0	0.1	76.6	1.3
C-2	28.0	28.7	0.7	26.3	1.7
C-3	35.8	36.2	0.4	34.6	1.2
C-4	86.6	84.8	1.8	85.1	1.5
C-5	180.8	184.0	3.2	183.1	2.3
C-6	41.5	43.6	2.1	44.1	2.6
C-7	172.0	168.3	3.7	168.2	3.8
C-8	114.5	115.7	1.2	118.7	4.2
C-9	24.6	24.4	0.2	23.5	1.1
C-10	25.9	24.5	1.4	22.9	3.0
C-11	18.1	17.6	0.5	22.7	4.6
MAE		1.39		2.48	
R^2		0.9990		0.9976	
DP4$^+$		100.00%		0.00%	

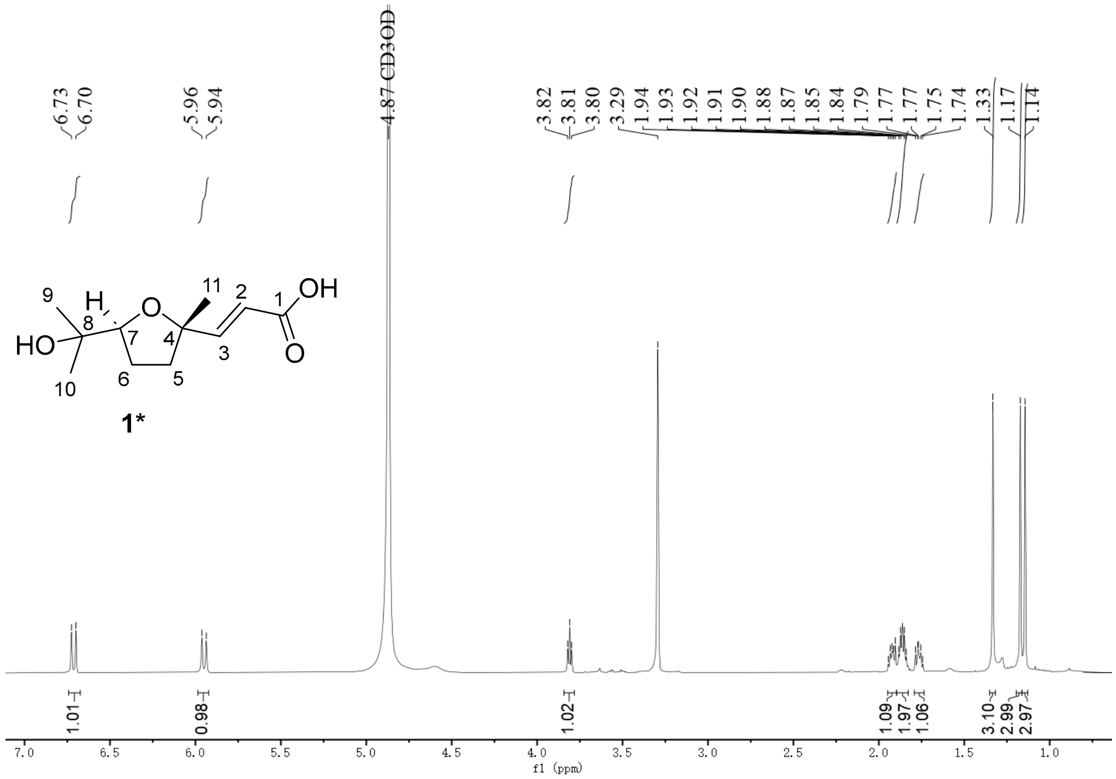

Figure S2. ^{1}H NMR (600 MHz) spectrum of compound **1** in CD_3OD

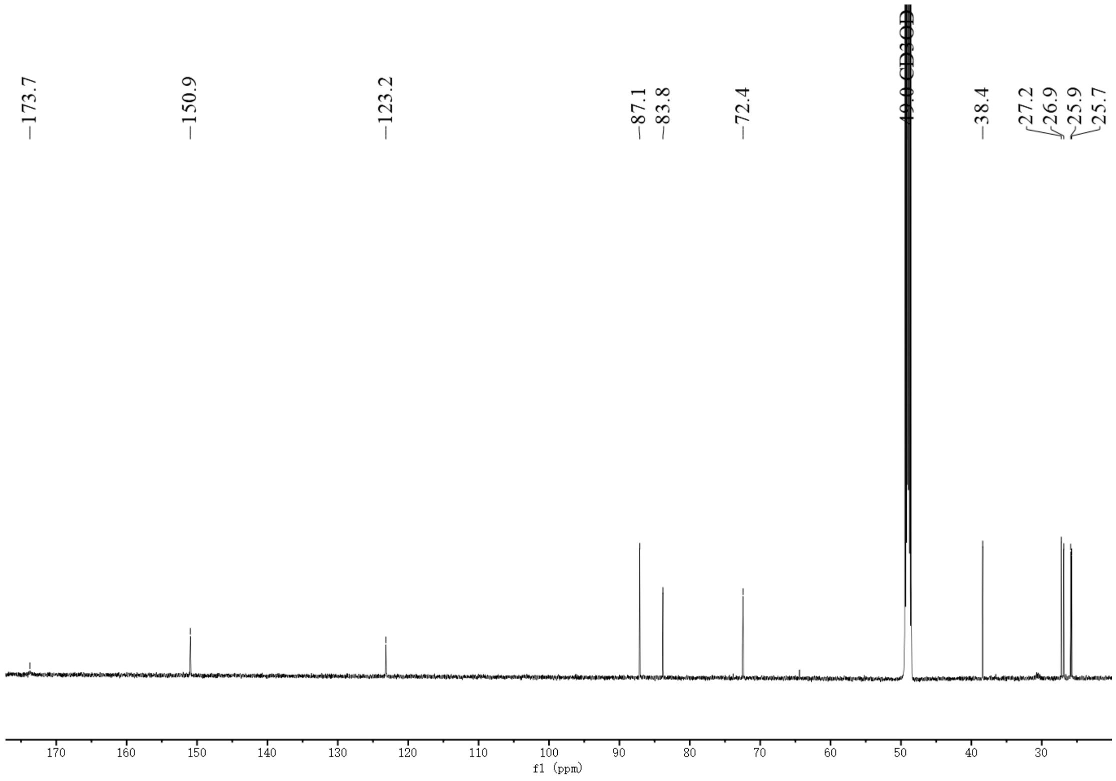

Figure S3. ^{13}C NMR (150 MHz) spectrum of compound **1** in CD_3OD

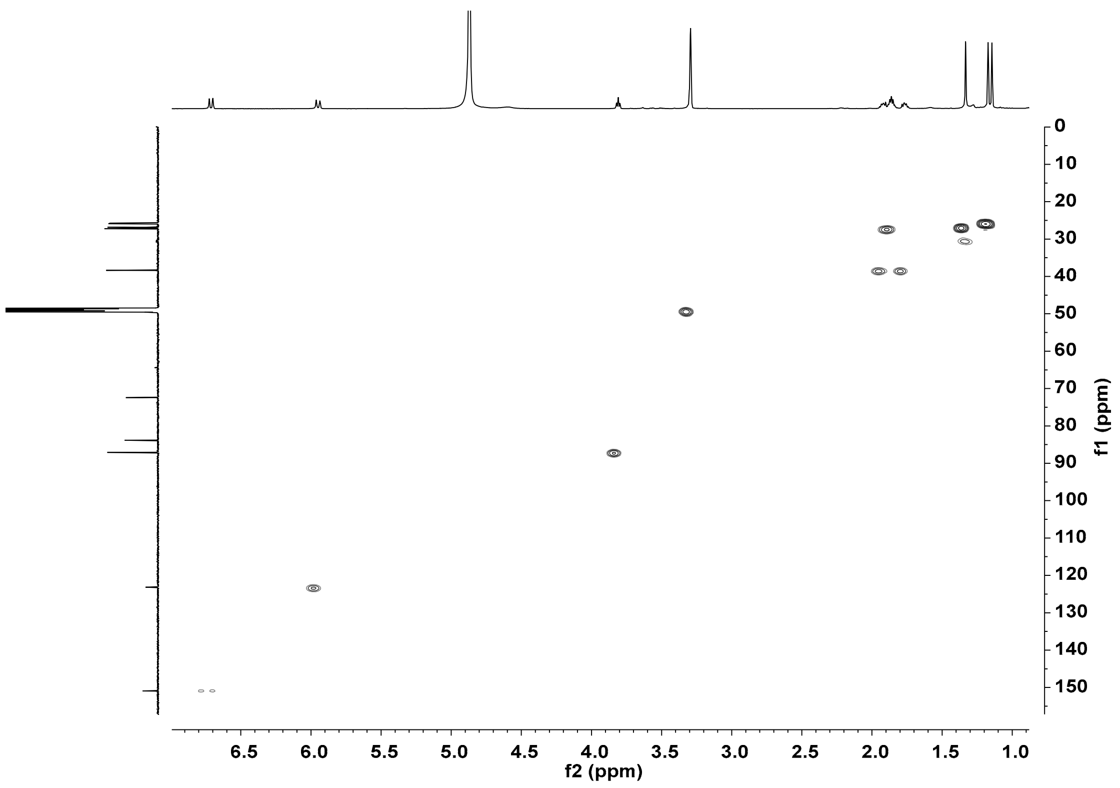

Figure S4. HSQC spectrum of compound **1** in CD_3OD

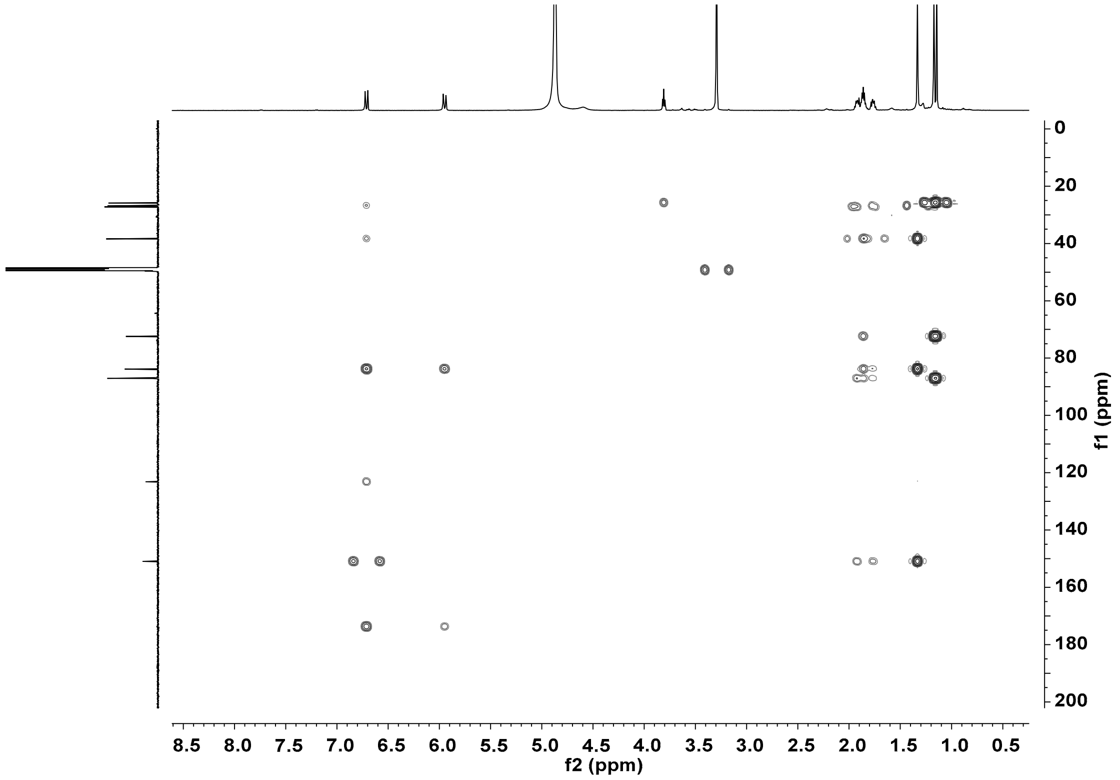

Figure S5. HMBC spectrum of compound **1** in CD_3OD

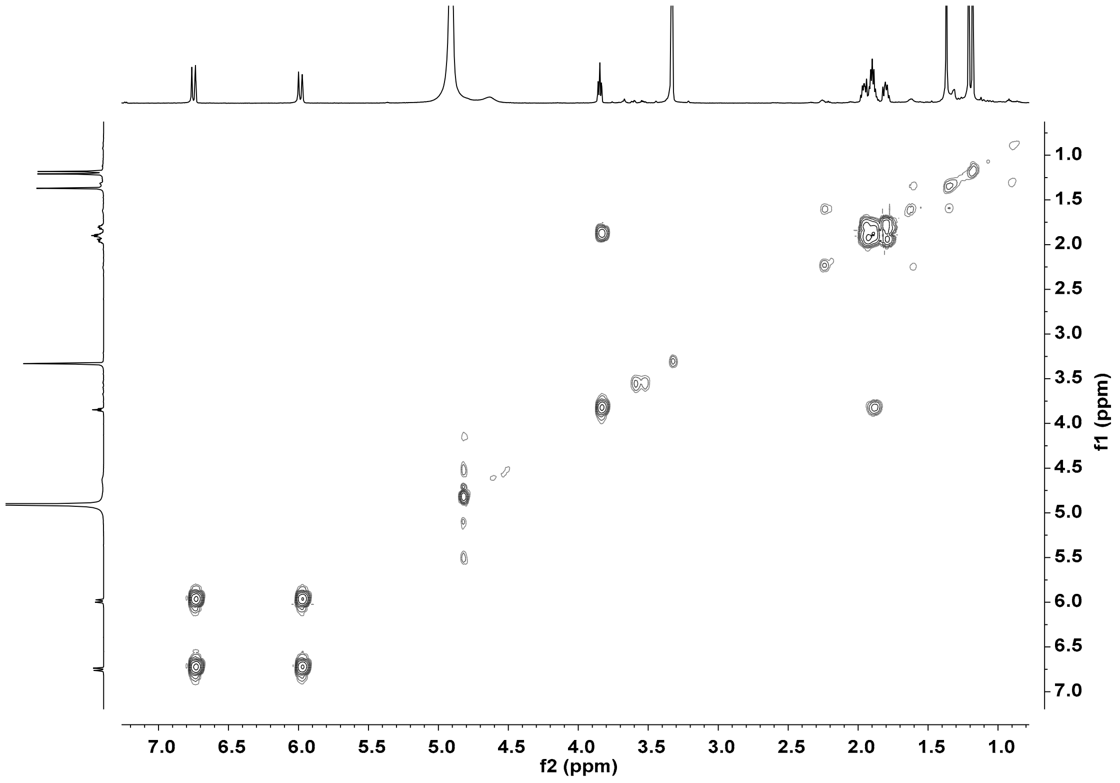

Figure S6. ^1H–^1H COSY spectrum of compound **1** in CD_3OD

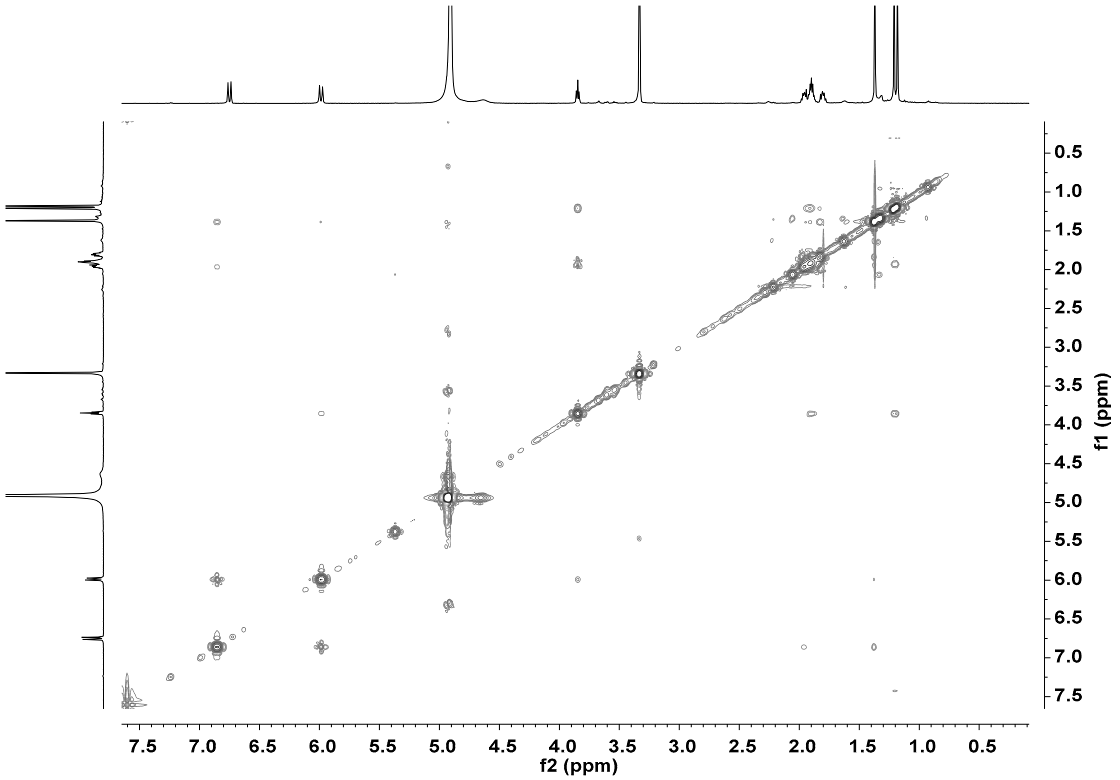

Figure S7. NOESY spectrum of compound **1** in CD_3OD

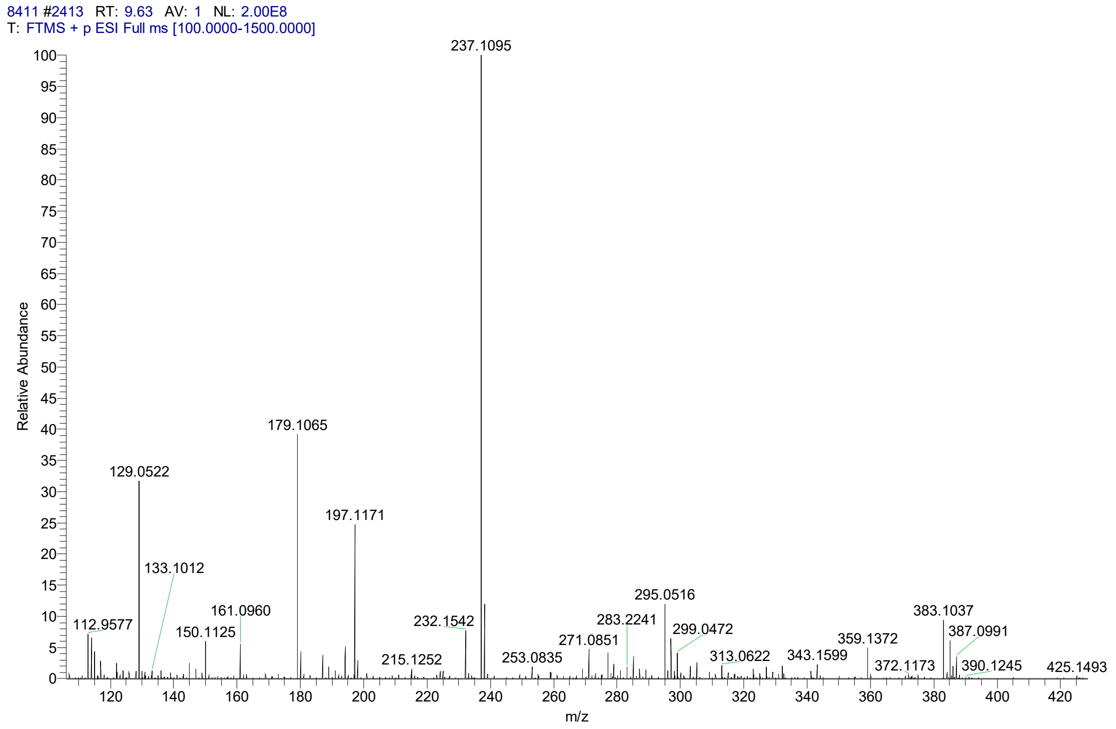

Figure S8. (+)-HRESIMS spectrum of compound **1**

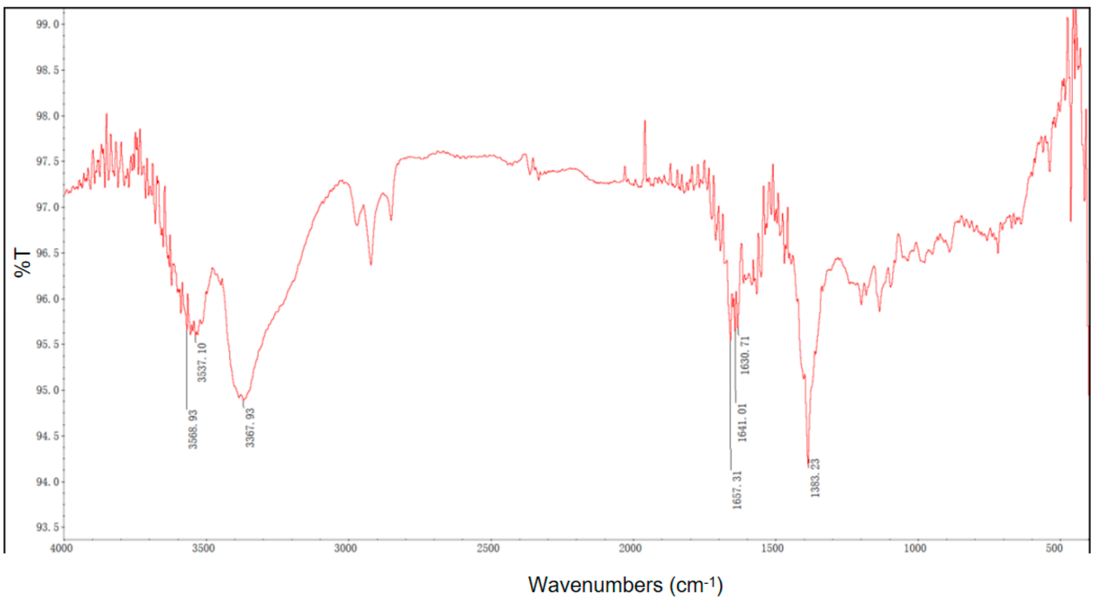

Figure S9. IR (KBr) spectrum of compound **1**

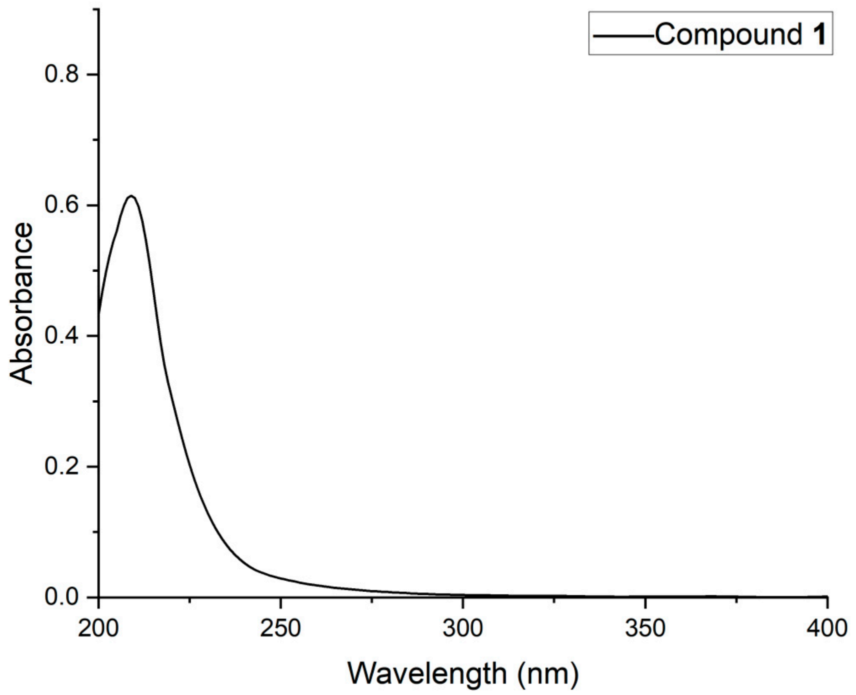

Figure S10. UV spectrum of compound **1**

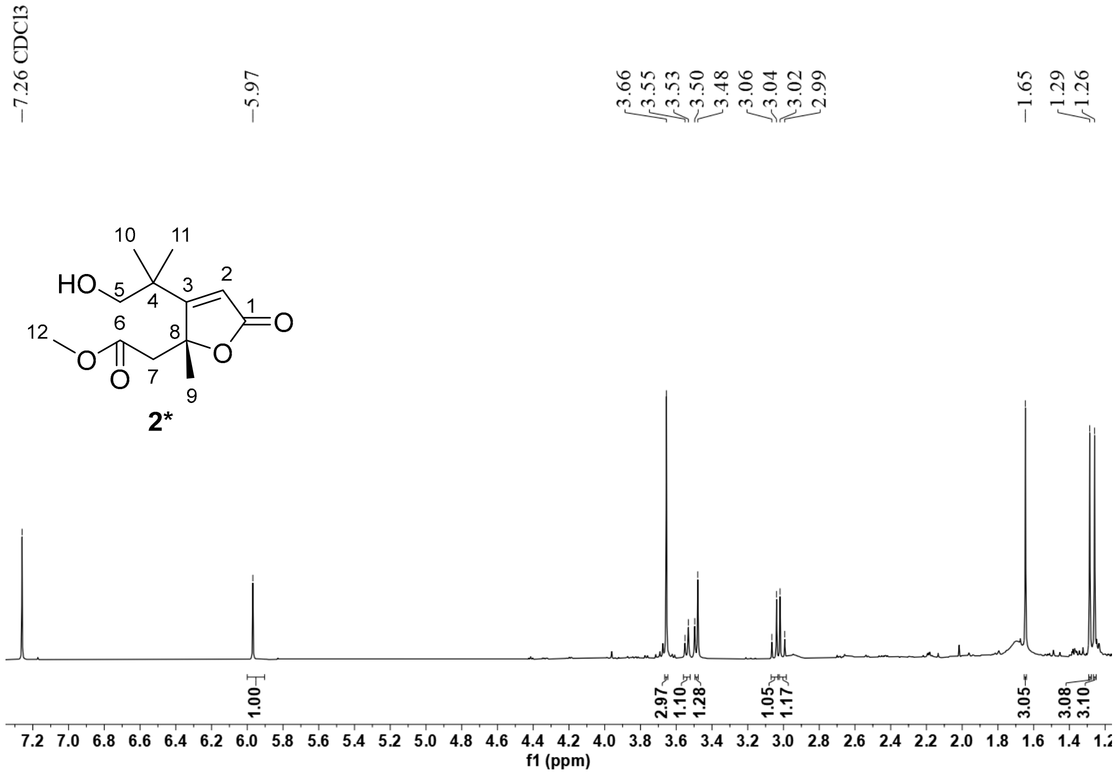

Figure S11. ^{1}H NMR (600 MHz) spectrum of compound **2** in $CDCl_3$

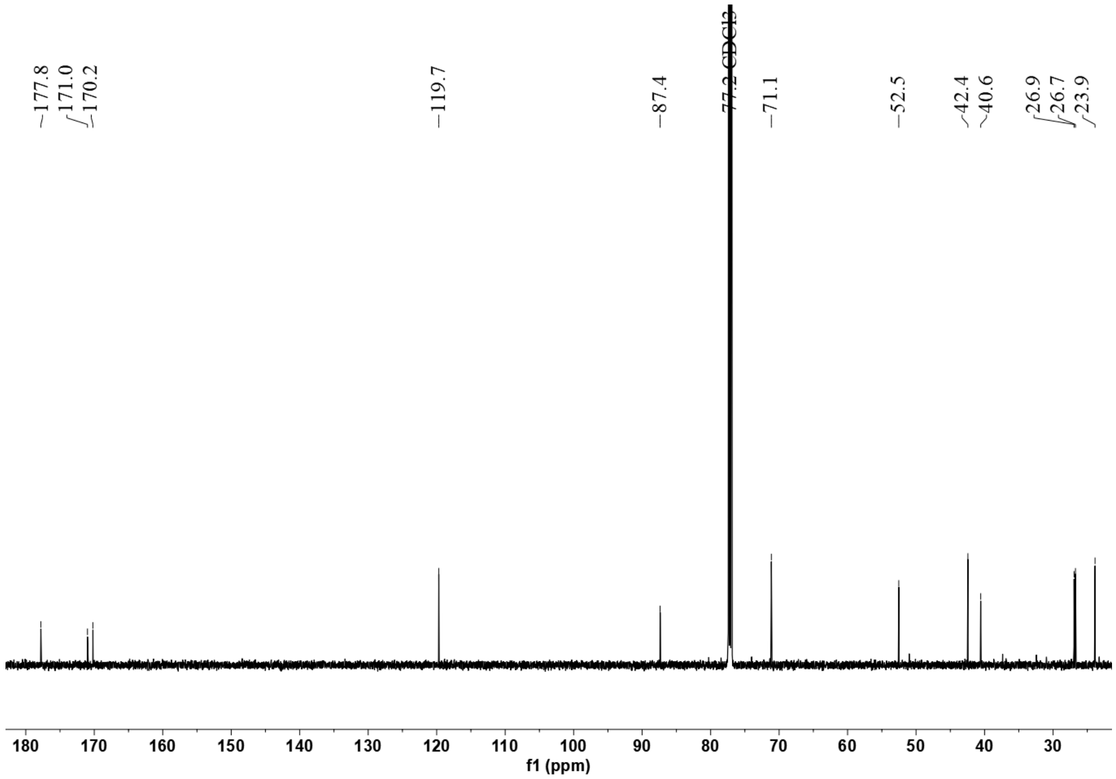

Figure S12. ^{13}C NMR (150 MHz) spectrum of compound **2** in $CDCl_3$

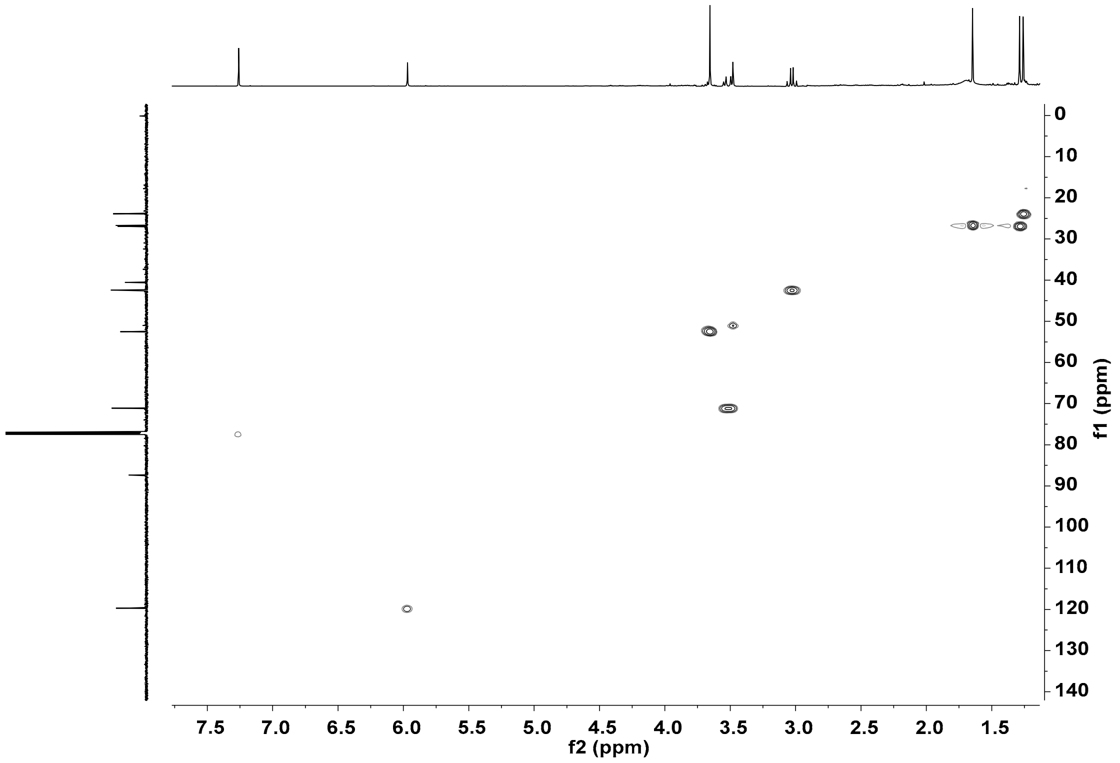

Figure S13. HSQC spectrum of compound **2** in $CDCl_3$

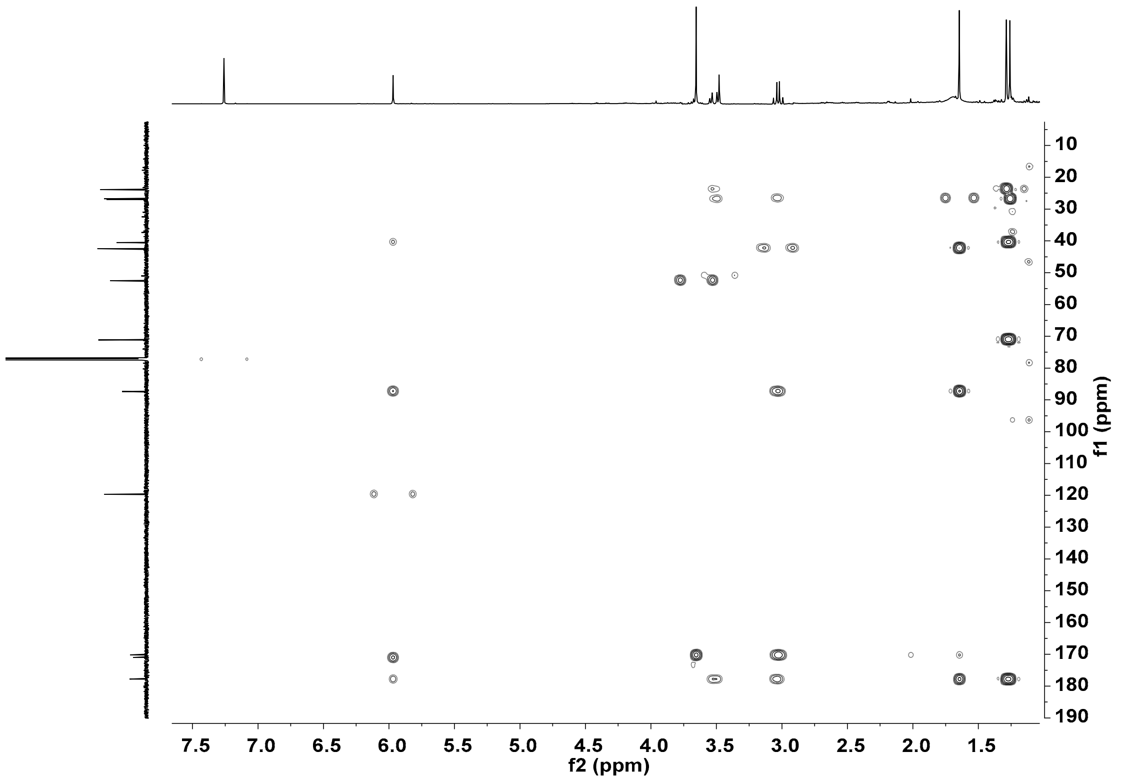

Figure S14. HMBC spectrum of compound **2** in $CDCl_3$

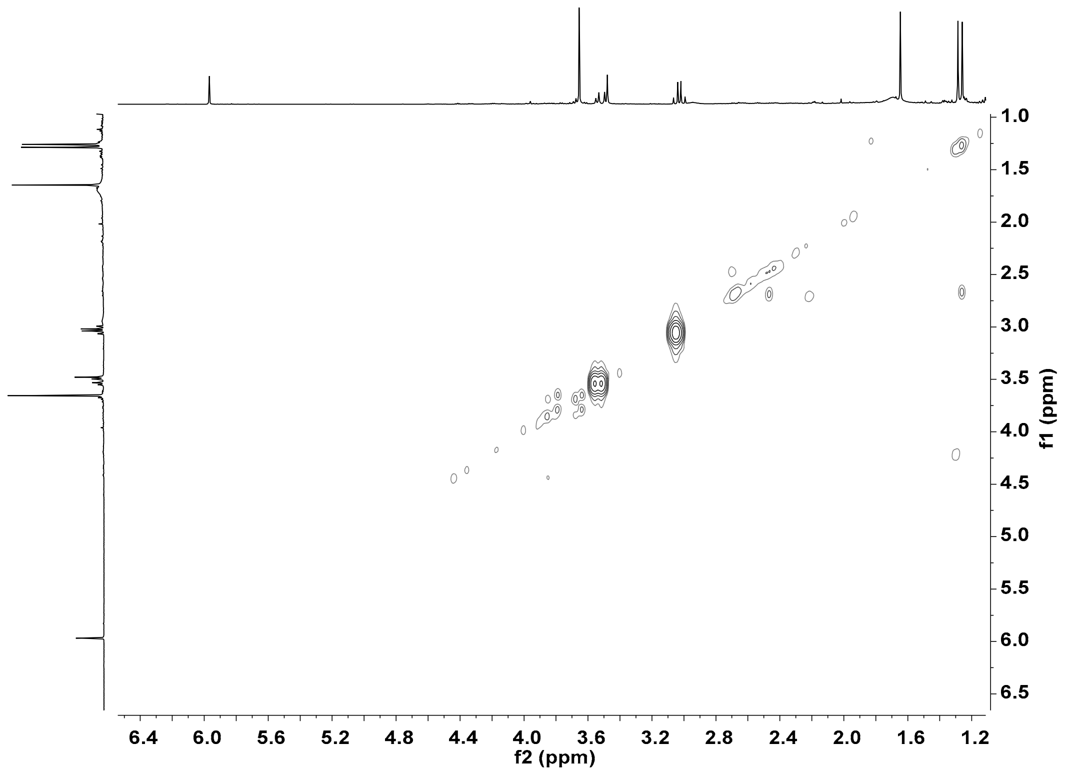

Figure S15. ^1H–^1H COSY spectrum of compound **2** in $CDCl_3$

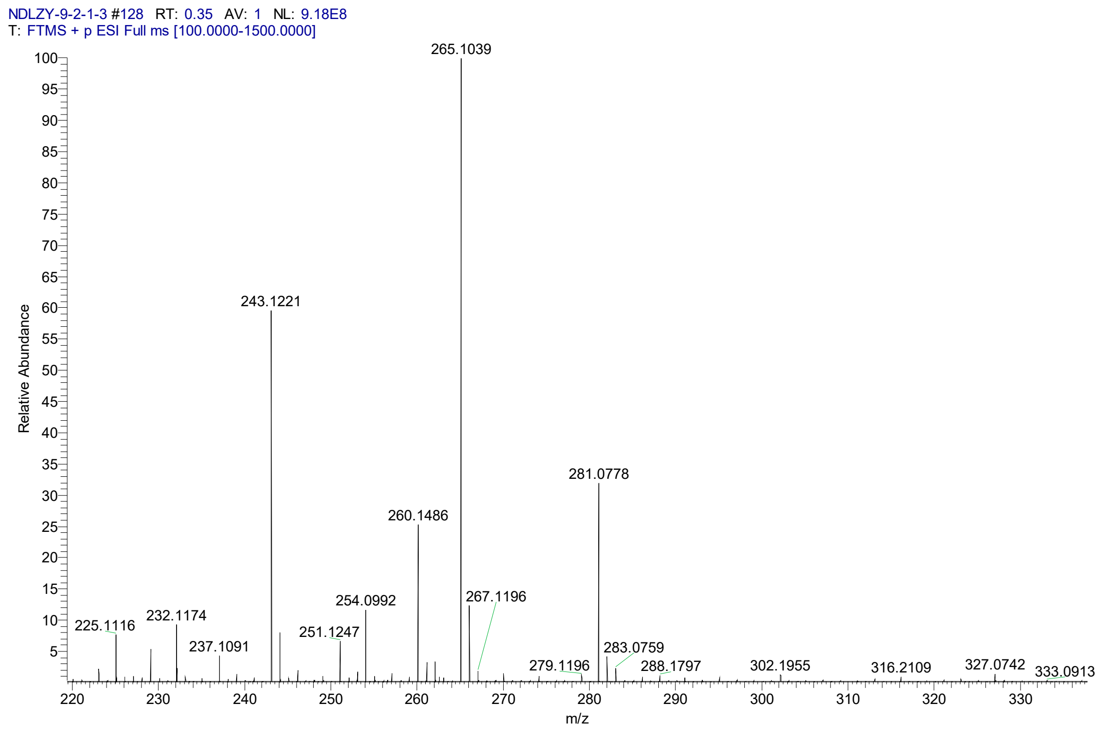

Figure S16. (+)-HRESIMS spectrum of compound **2**

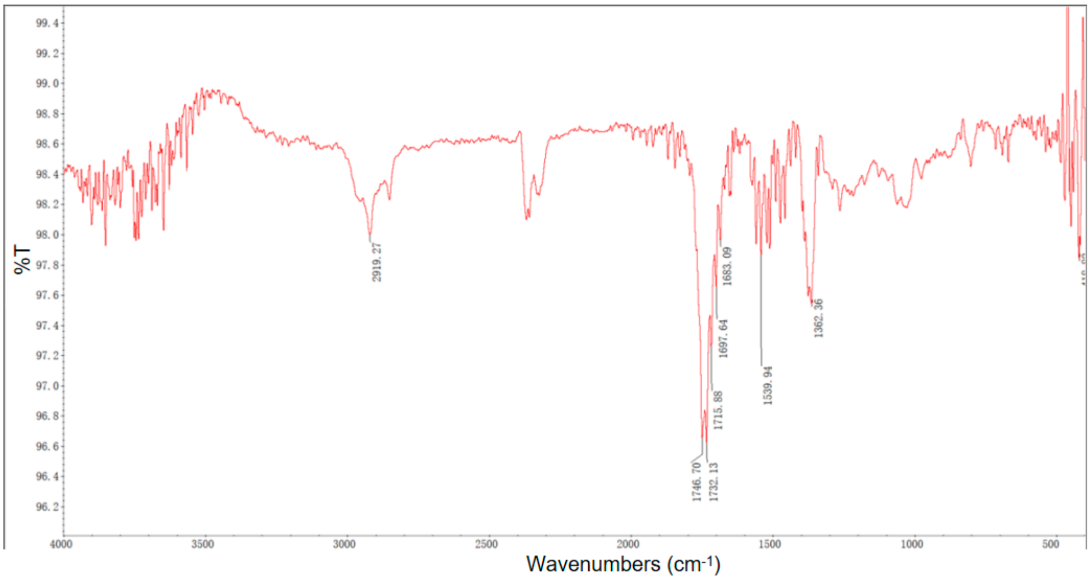

Figure S17. IR (KBr) spectrum of compound **2**

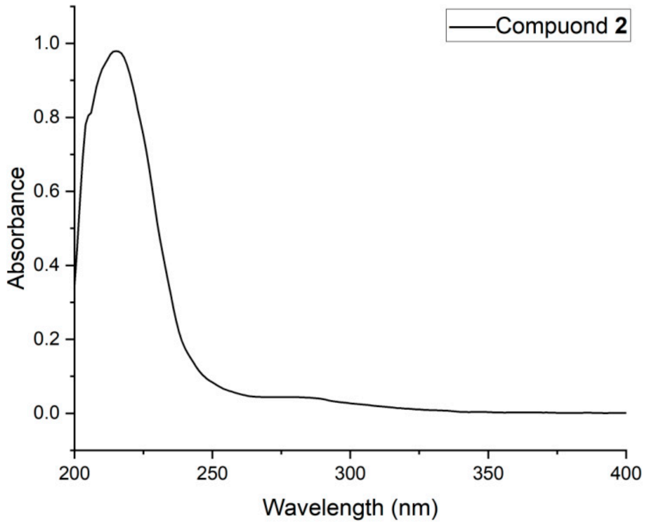

Figure S18. UV spectrum of compound **2**

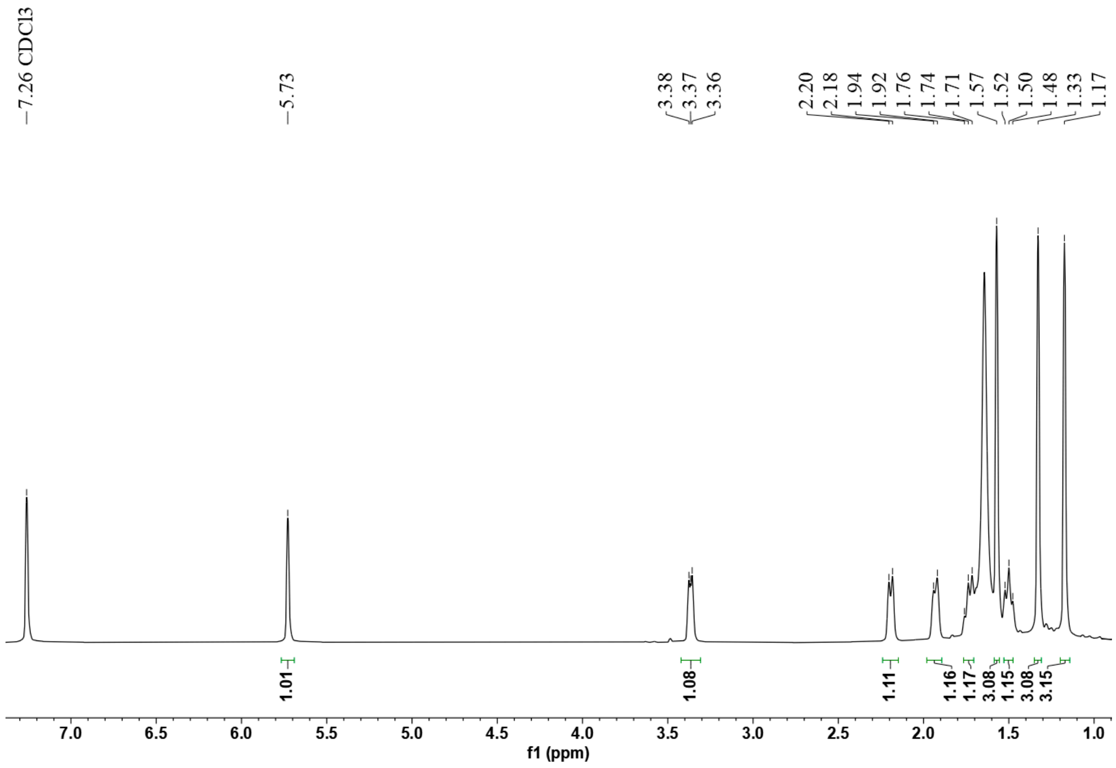

Figure S19. ^{1}H NMR (600 MHz) spectrum of compound **3** in $CDCl_3$

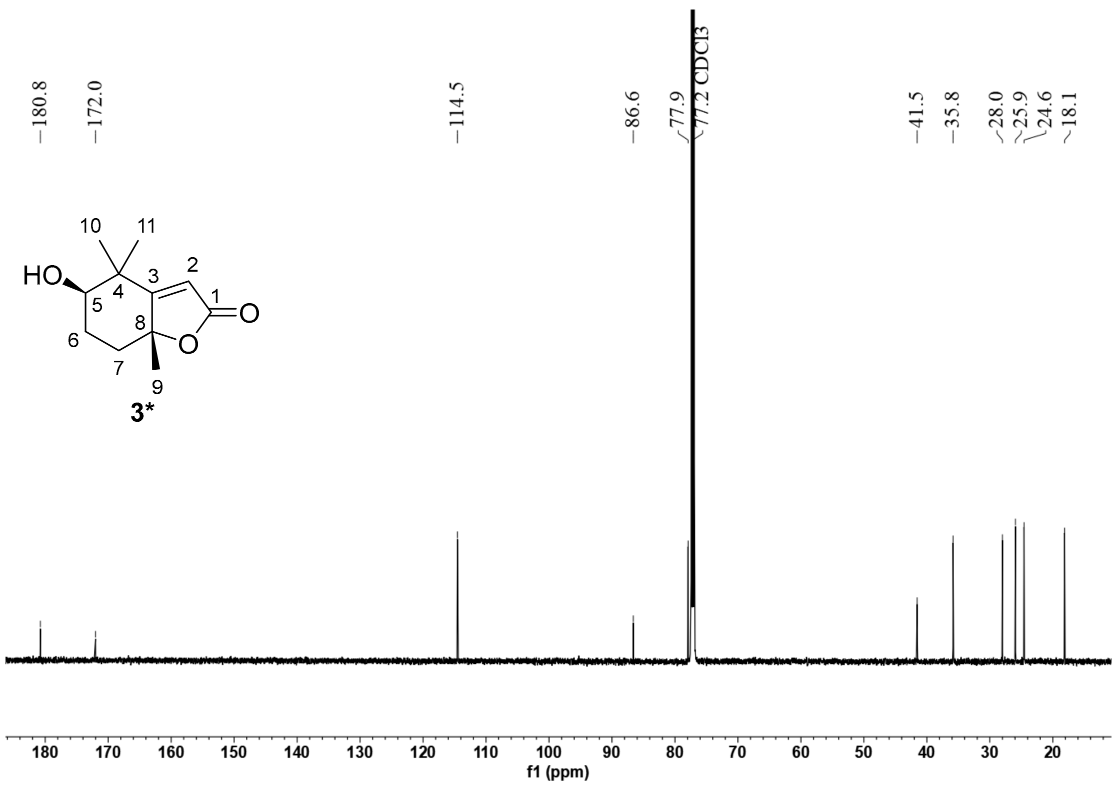

Figure S20. ^{13}C NMR (150 MHz) spectrum of compound **3** in $CDCl_3$

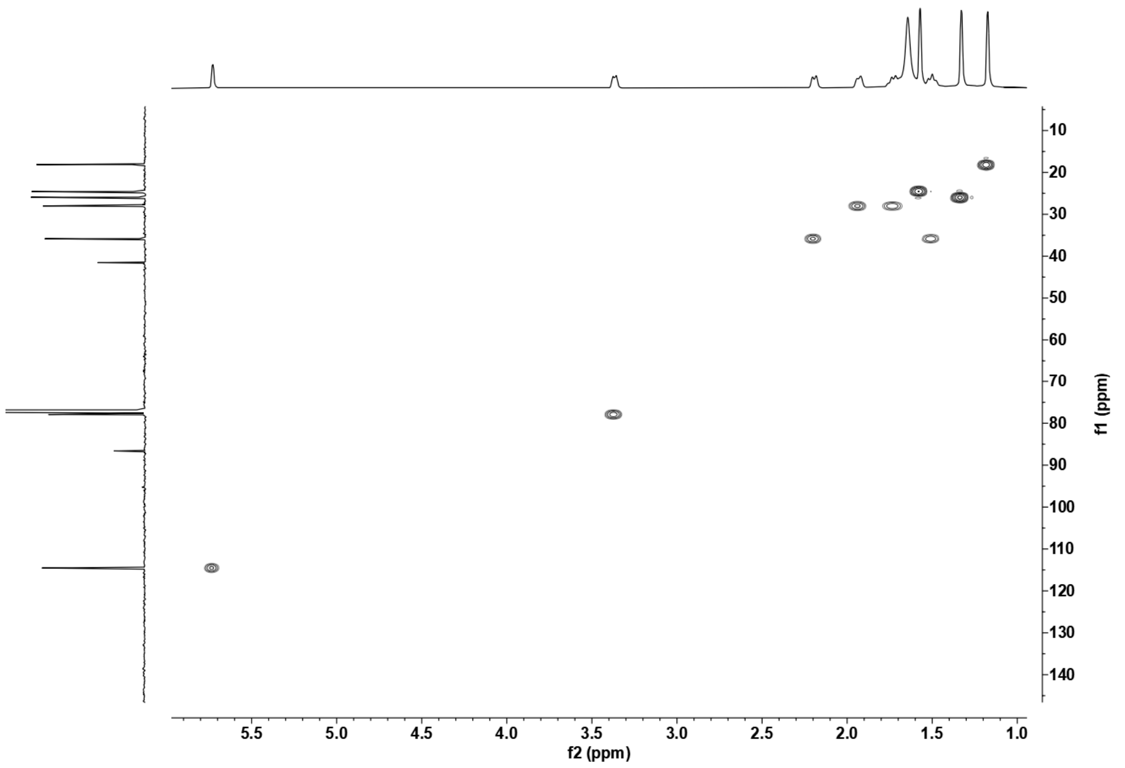

Figure S21. HSQC spectrum of compound **3** in $CDCl_3$

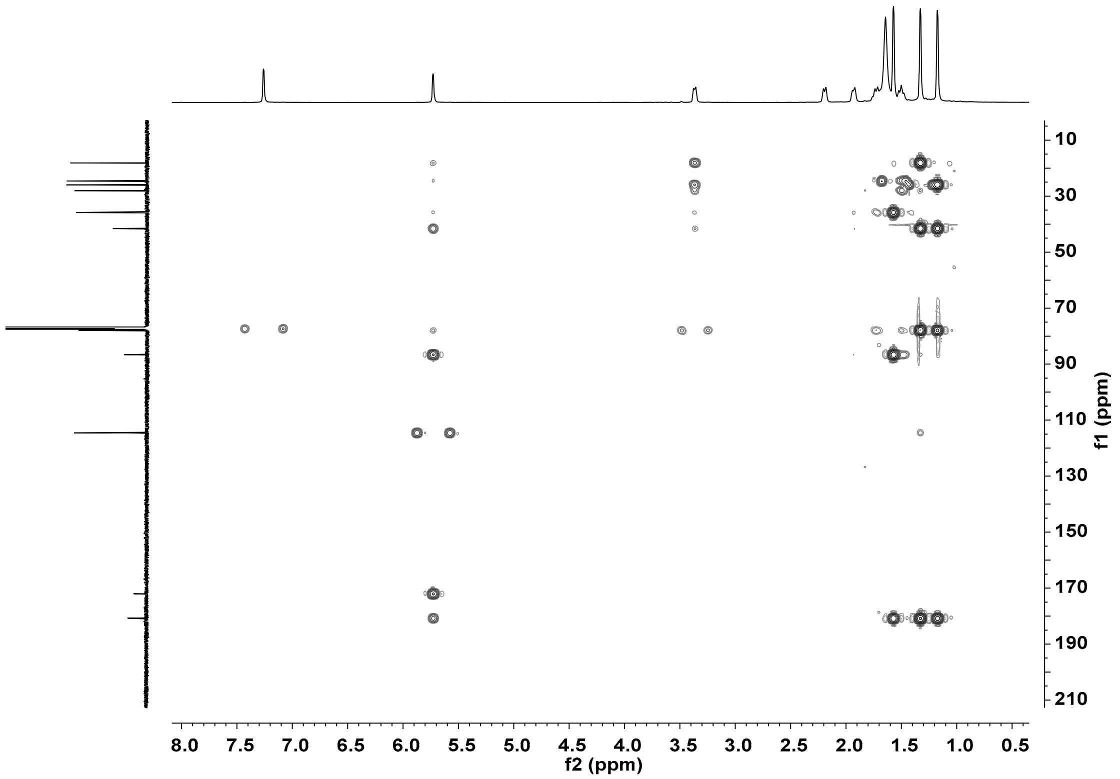

Figure S22. HMBC spectrum of compound **3** in $CDCl_3$

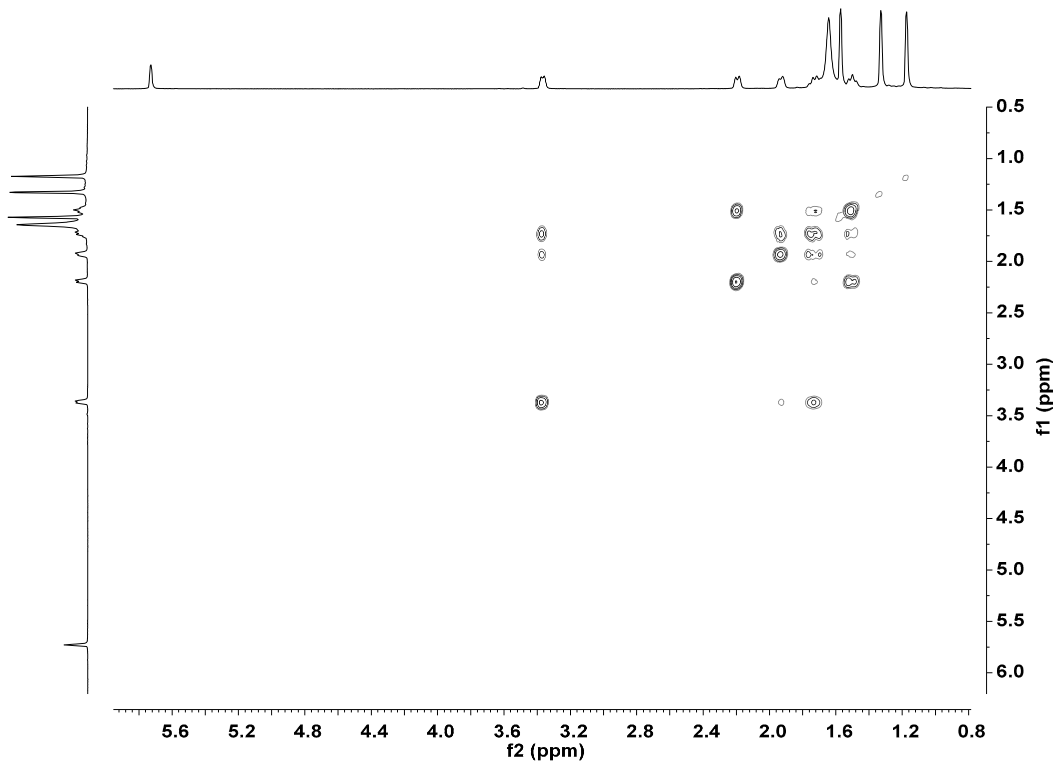

Figure S23. ^{1}H–^{1}H COSY spectrum of compound **3** in $CDCl_3$

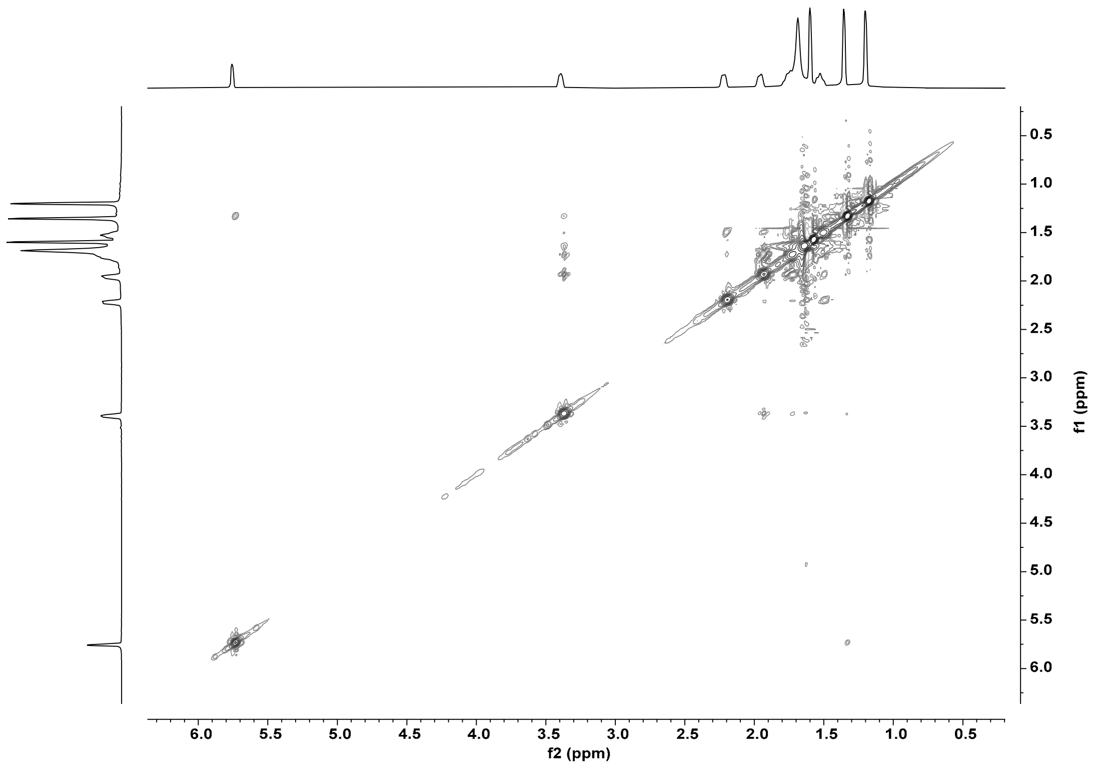

Figure S24. NOESY spectrum of compound **3** in $CDCl_3$

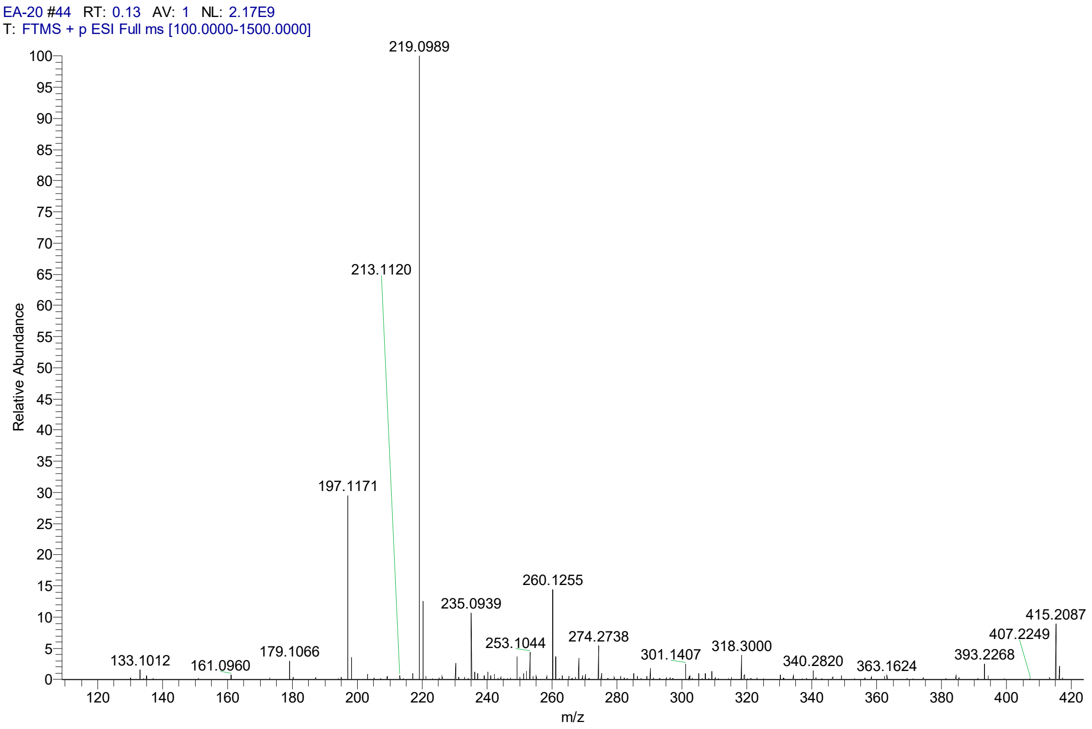

Figure S25. (+)-HRESIMS spectrum of compound **3**

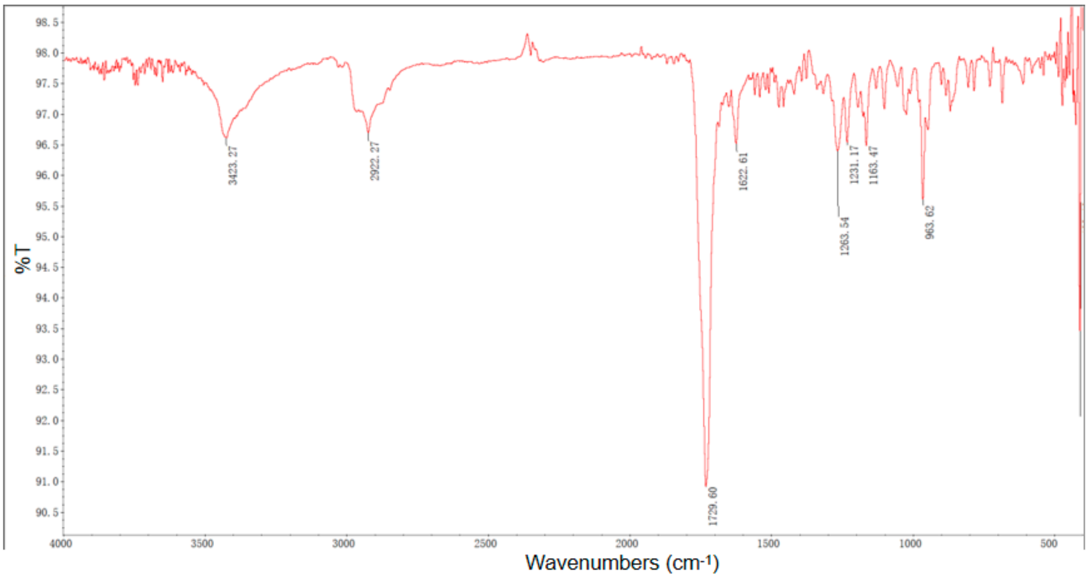

Figure S26. IR (KBr) spectrum of compound **3**

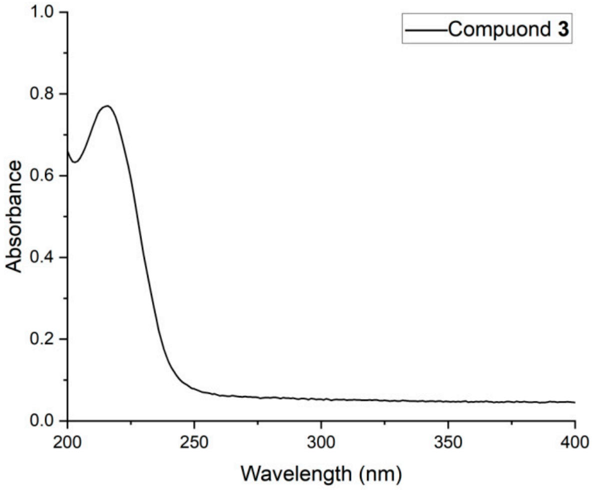

Figure S27. UV spectrum of compound **3**

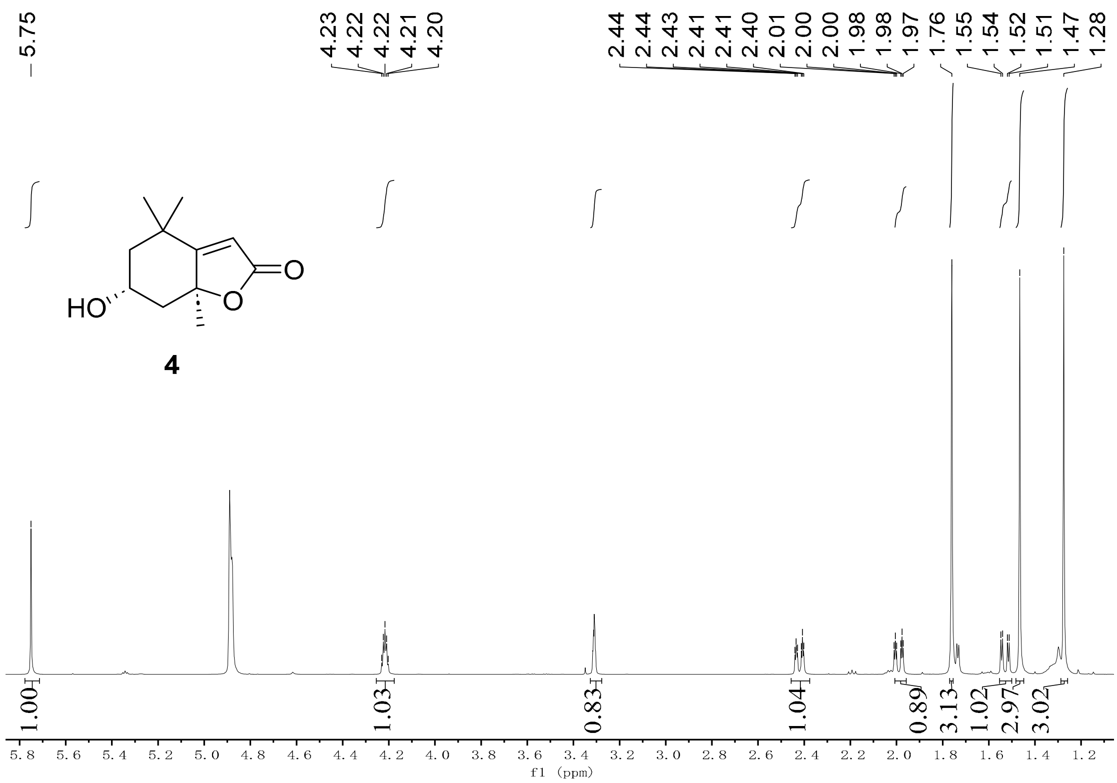

Figure S28. ^{1}H NMR (500 MHz) spectrum of compound **4** in CD_3OD

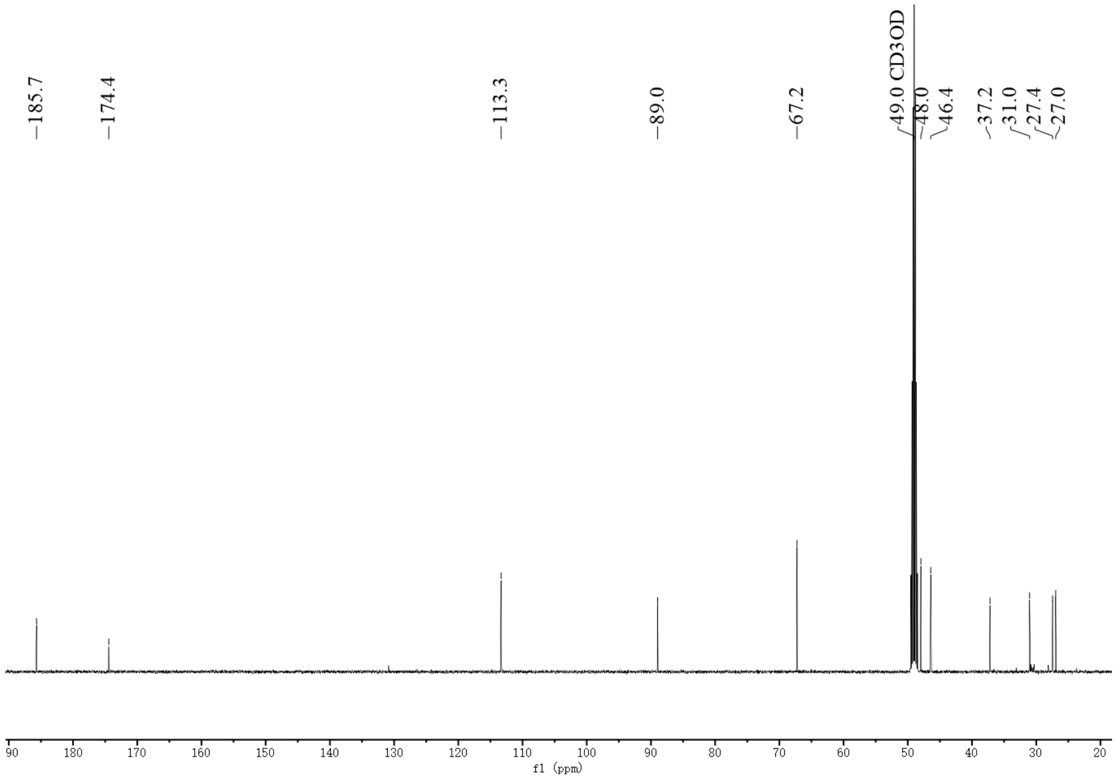

Figure S29. ^{13}C NMR (125 MHz) spectrum of compound **4** in CD_3OD

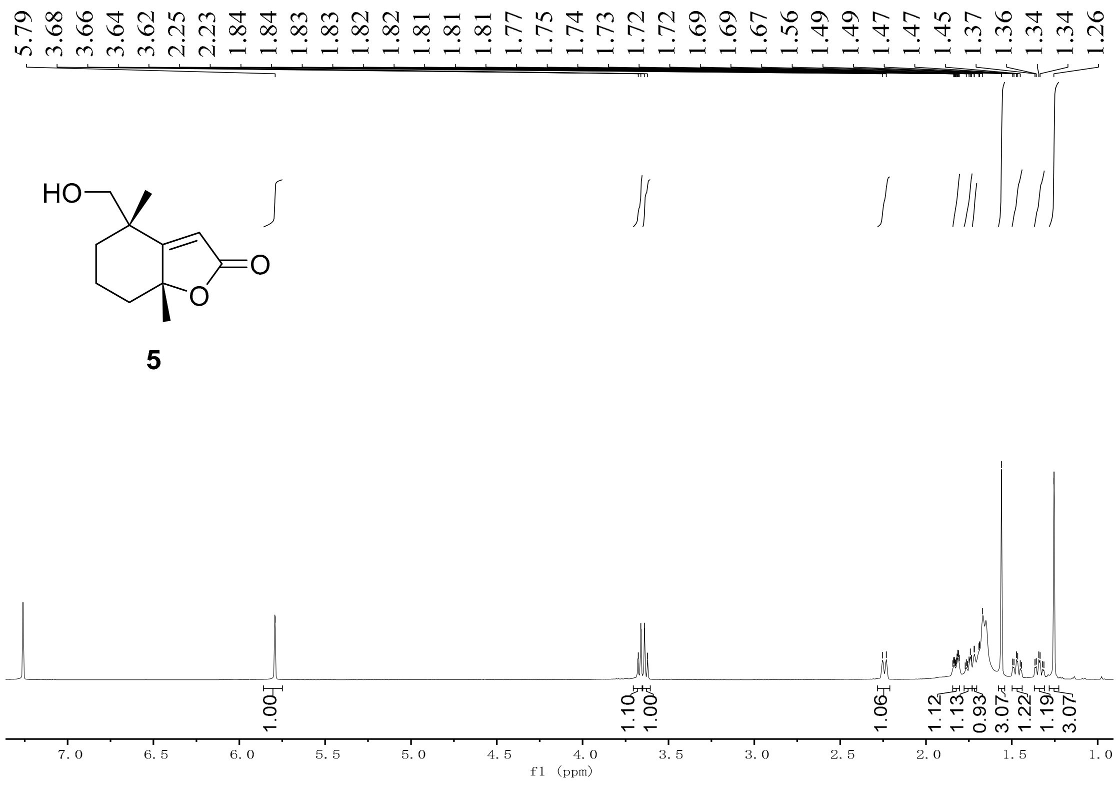

Figure S30. ^{1}H NMR (600 MHz) spectrum of compound **5** in $CDCl_3$

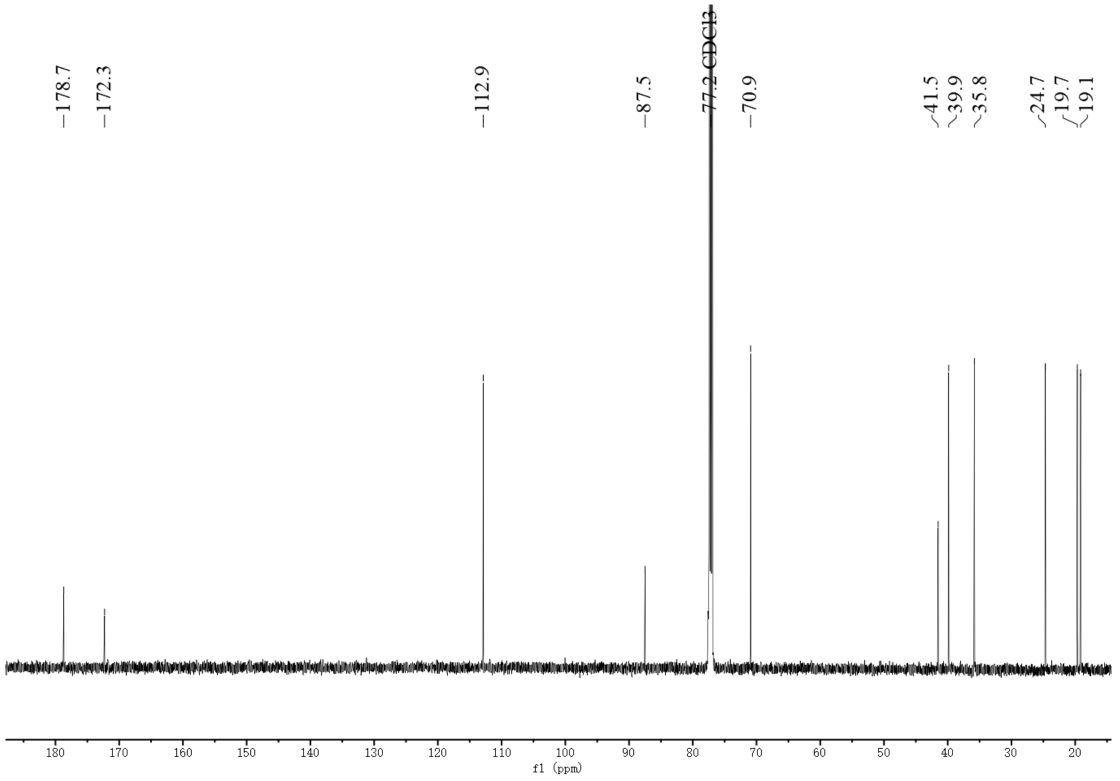

Figure S31. ^{13}C NMR (150 MHz) spectrum of compound **5** in $CDCl_3$

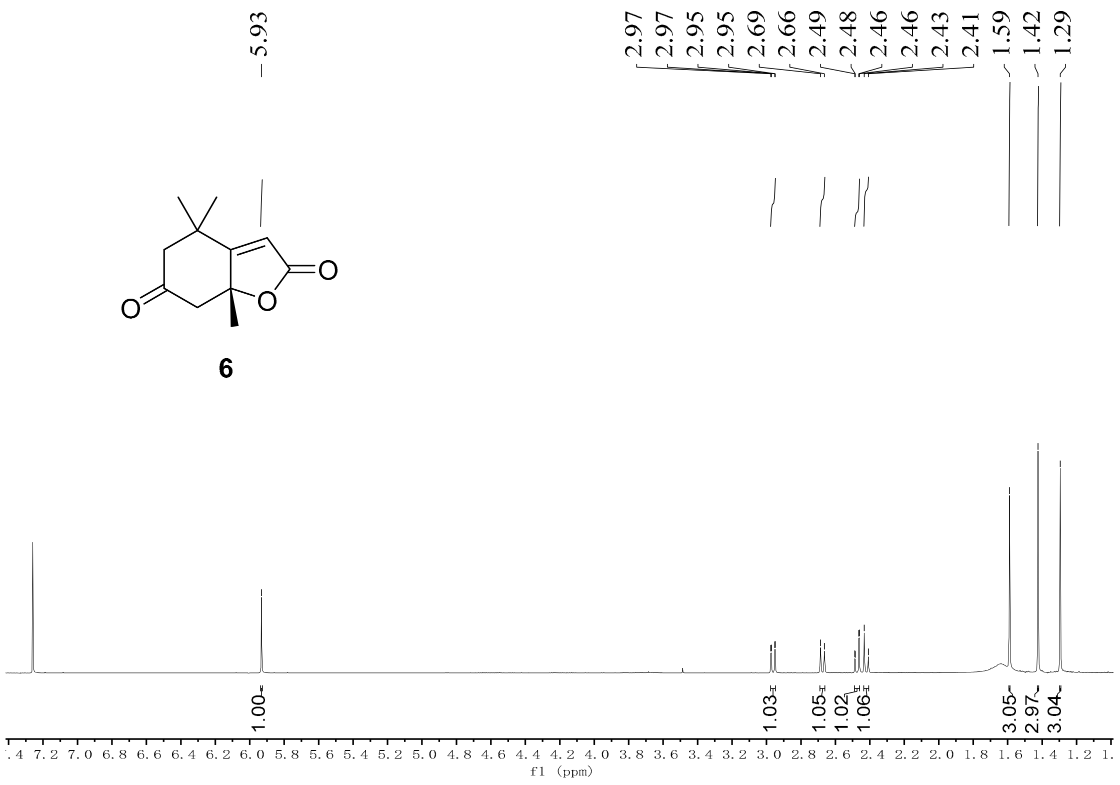

Figure S32. ^{1}H NMR (600 MHz) spectrum of compound **6** in $CDCl_3$

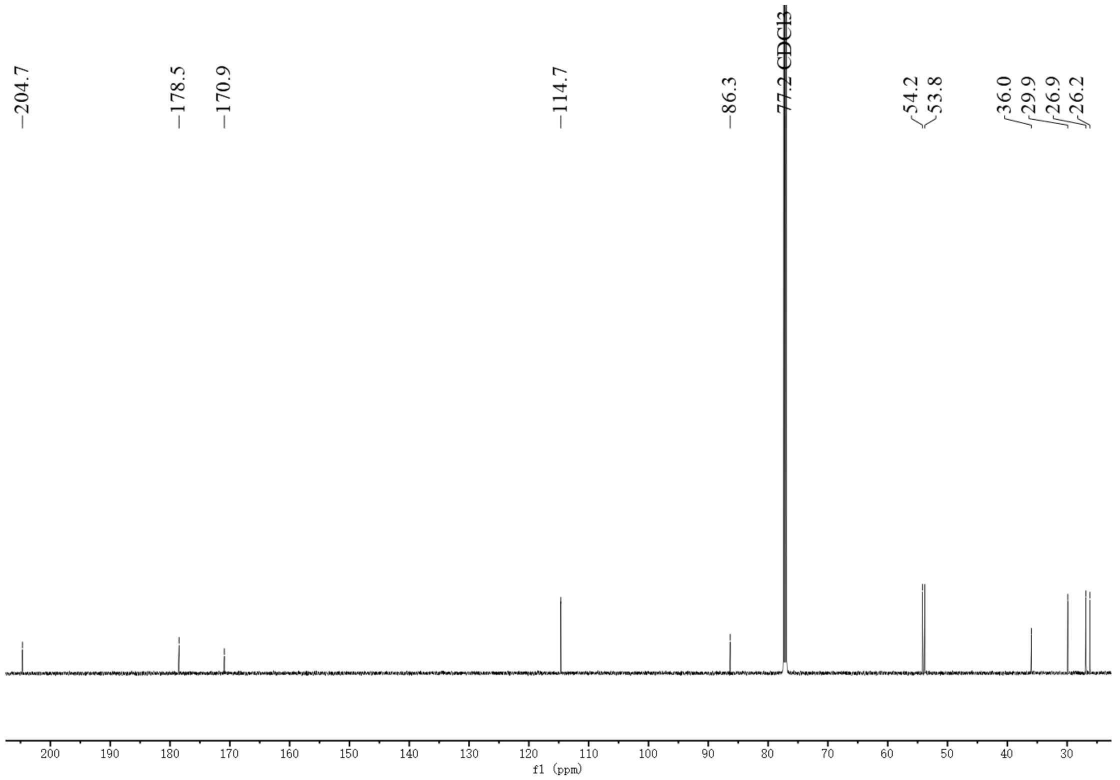

Figure S33. ^{13}C NMR (150 MHz) spectrum of compound **6** in $CDCl_3$

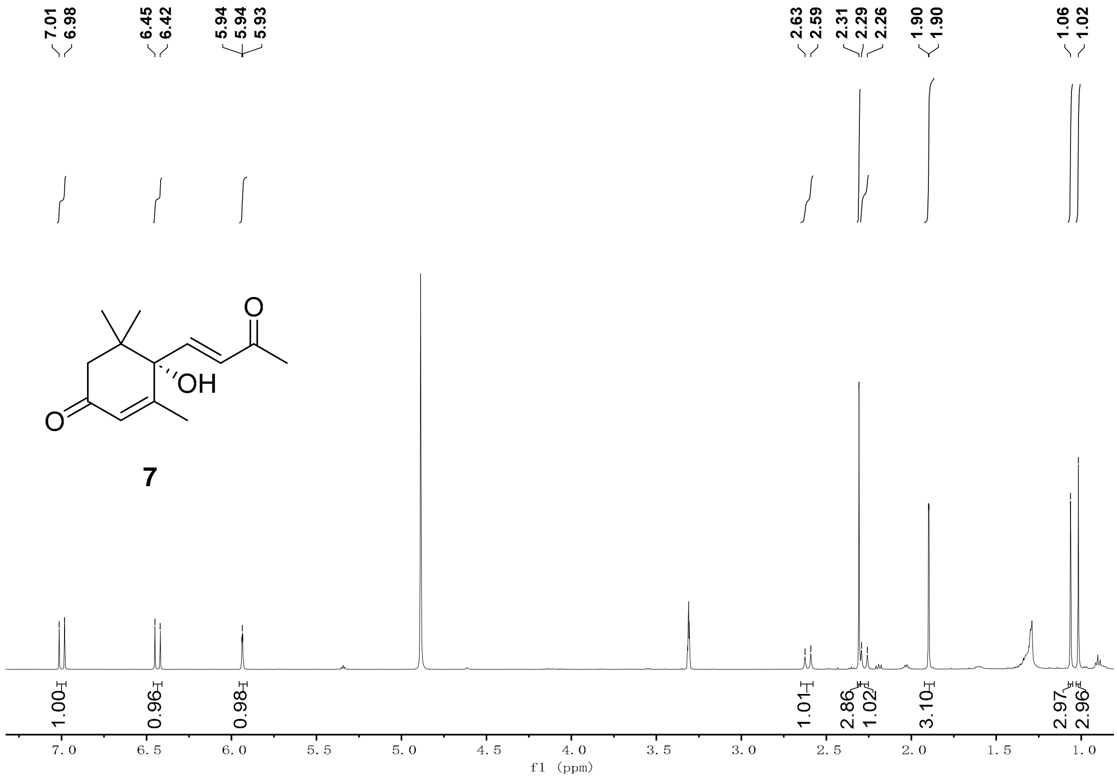

Figure S34. ^{1}H NMR (600 MHz) spectrum of compound **7** in CD_3OD

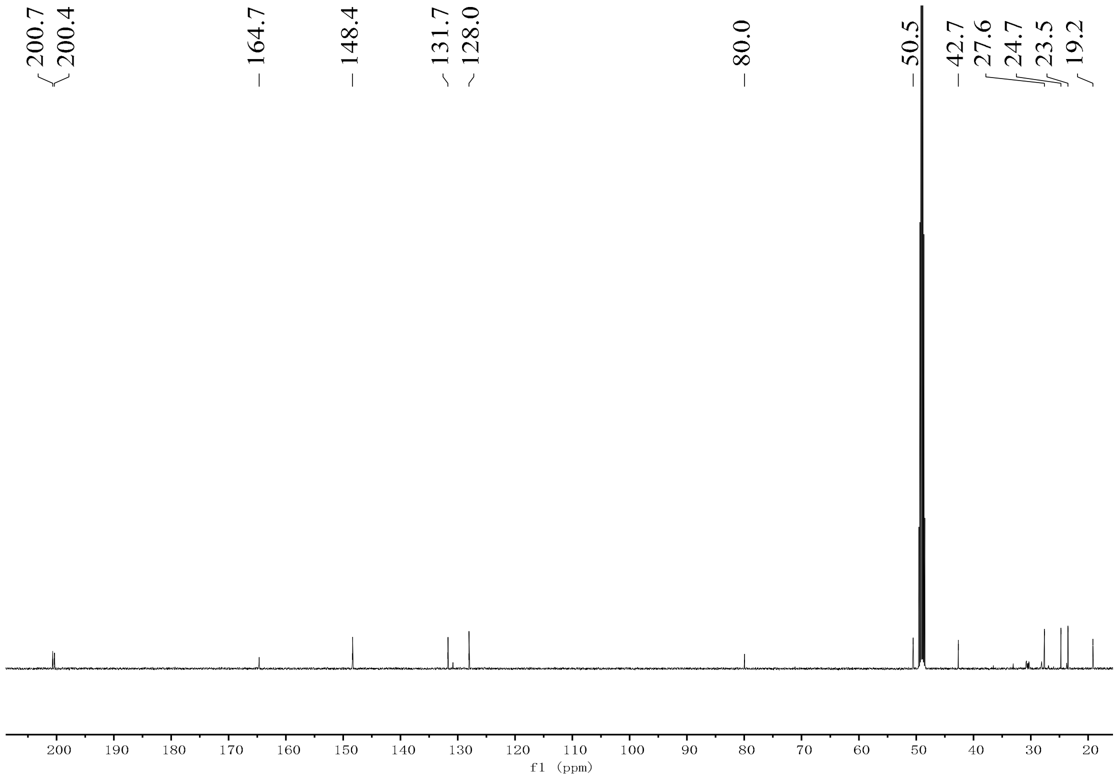

Figure S35. ^{13}C NMR (150 MHz) spectrum of compound **7** in CD_3OD

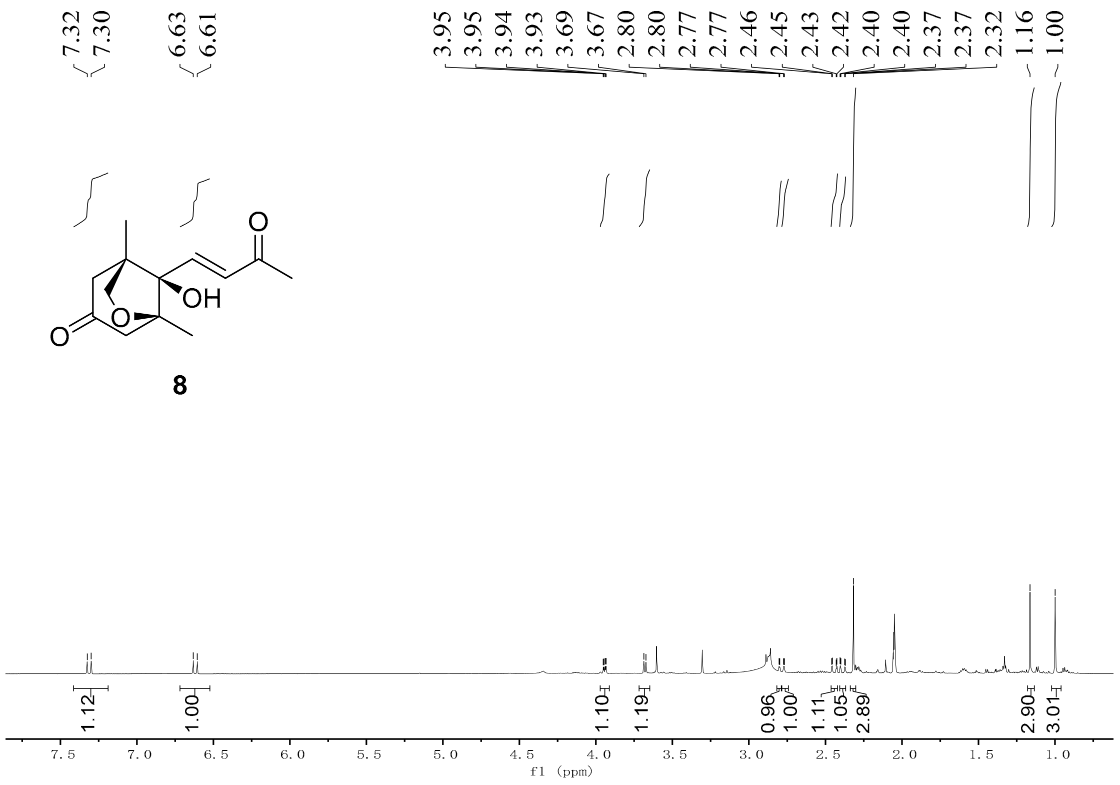

Figure S36. ^{1}H NMR (600 MHz) spectrum of compound **8** in Acetone-d_6

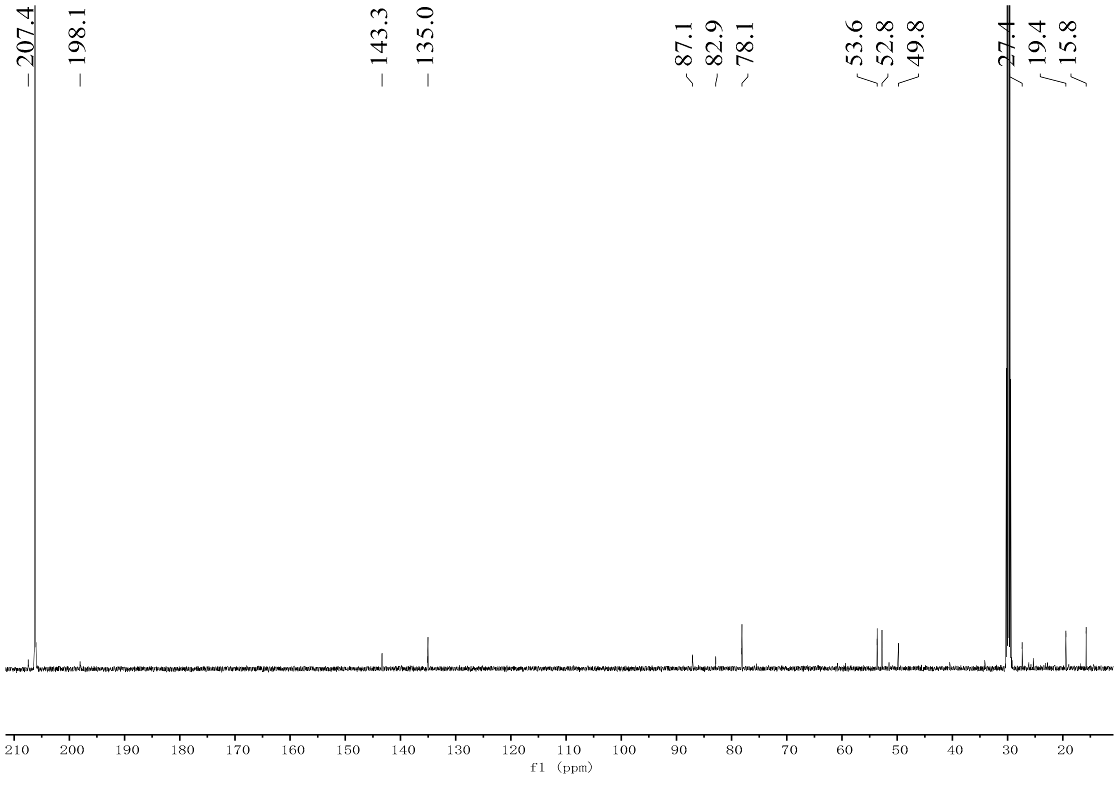

Figure S37. ^{13}C NMR (150 MHz) spectrum of compound **8** in Acetone-d_6

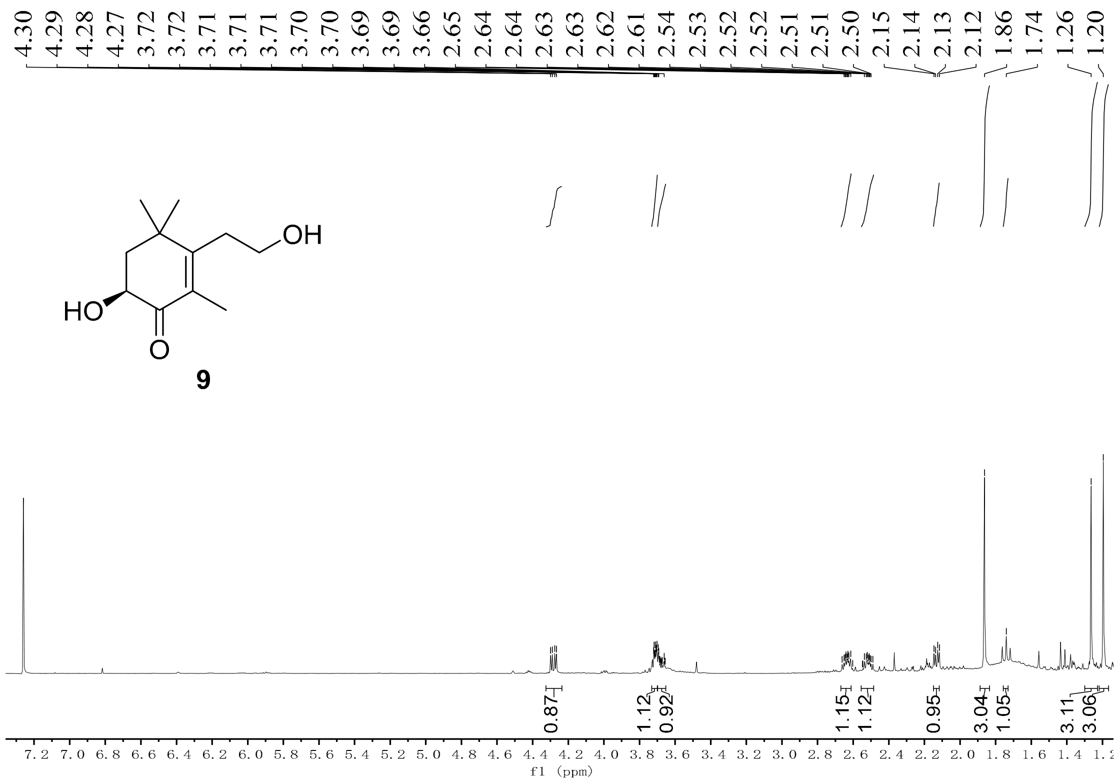

Figure S38. ^{1}H NMR (600 MHz) spectrum of compound **9** in $CDCl_3$

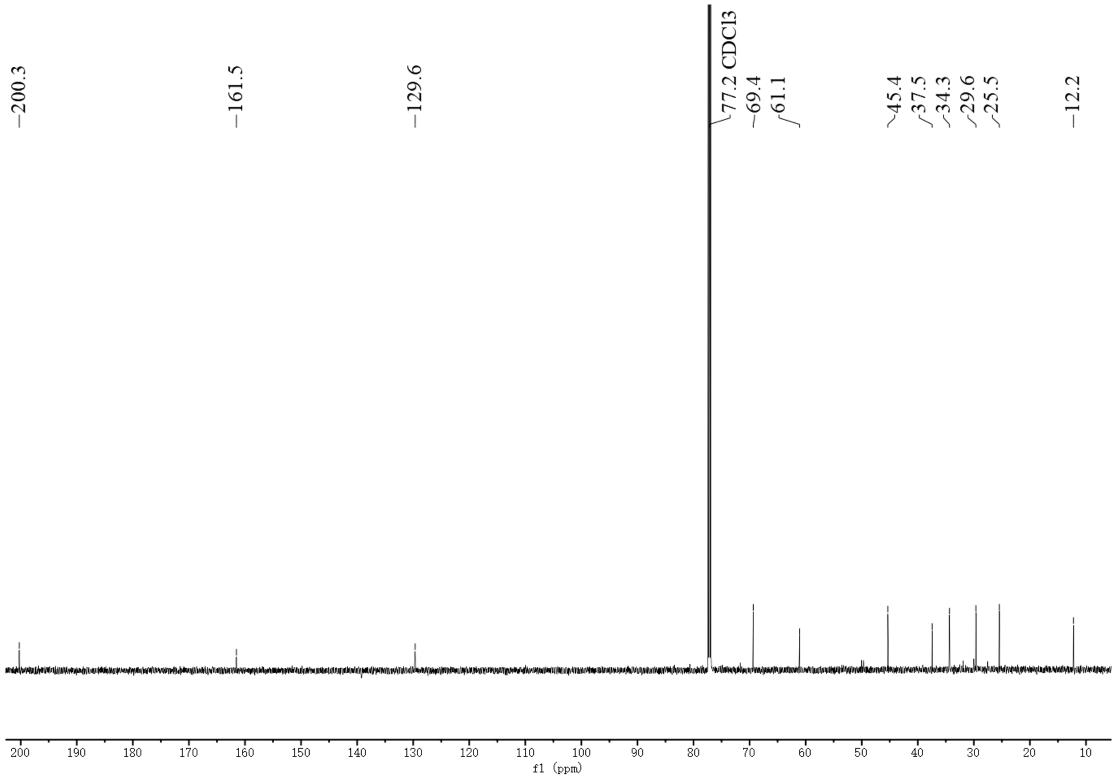

Figure S39. ^{13}C NMR (150 MHz) spectrum of compound **9** in $CDCl_3$

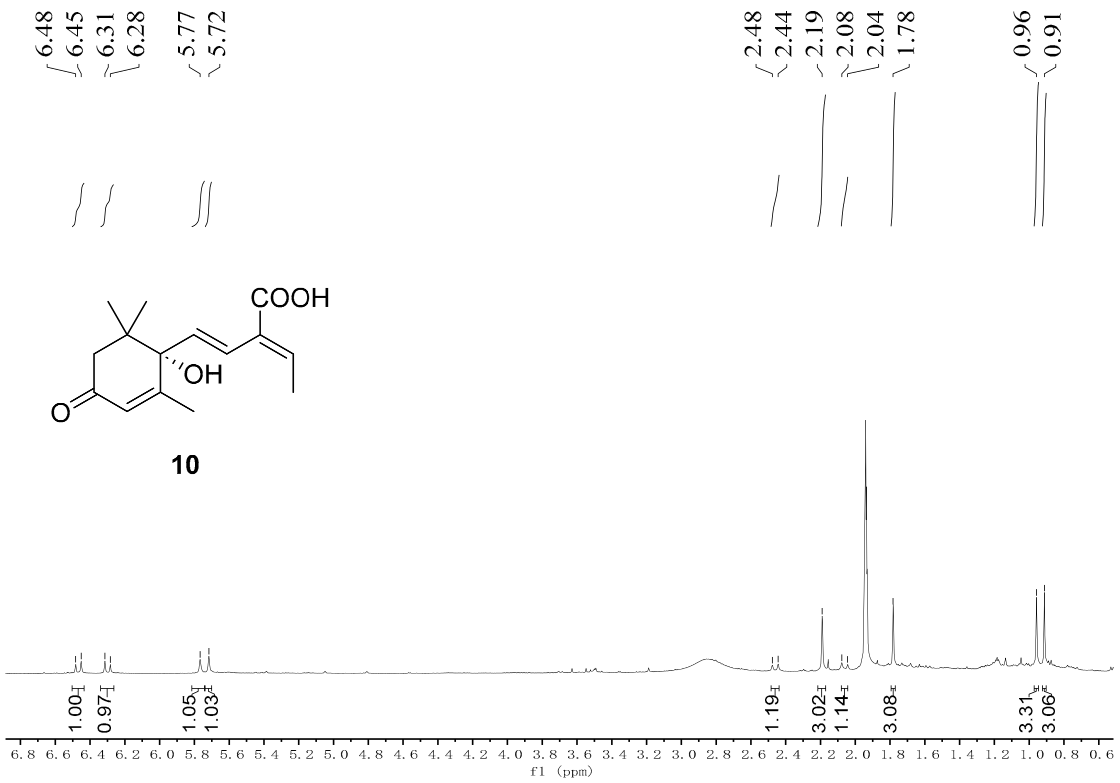

Figure S40. ^{1}H NMR (500 MHz) spectrum of compound **10** in Acetone-d_6

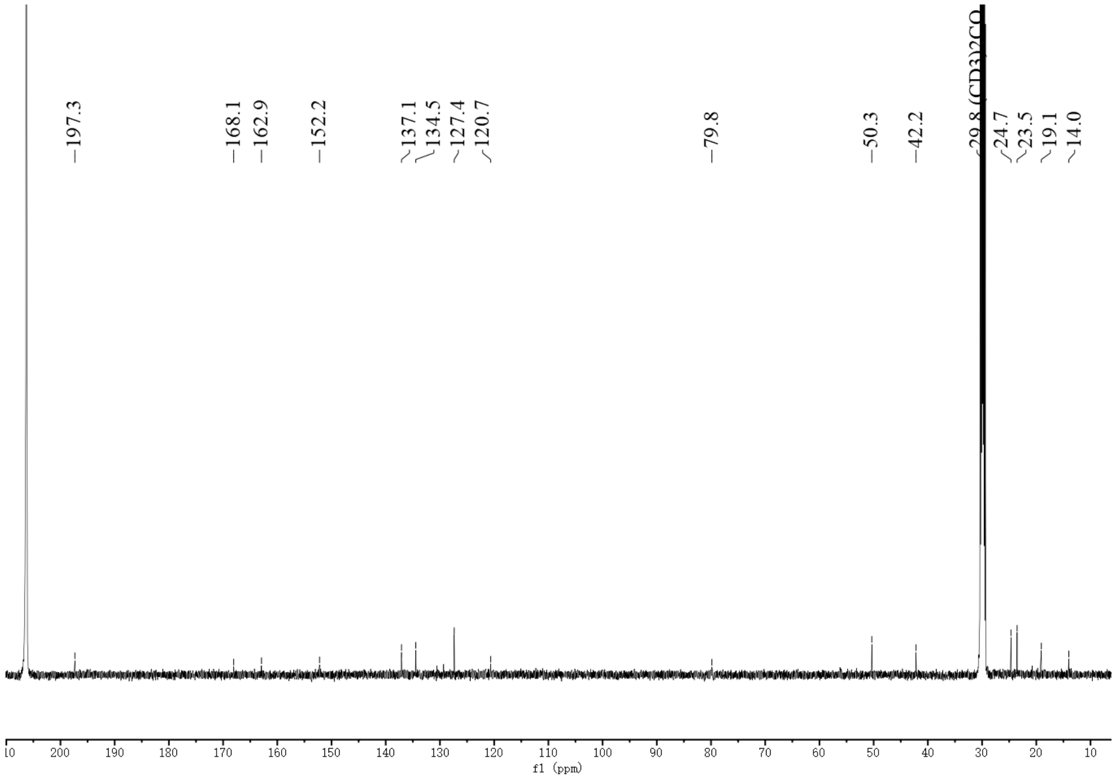

Figure S41. ^{13}C NMR (125 MHz) spectrum of compound **10** in Acetone-d_6

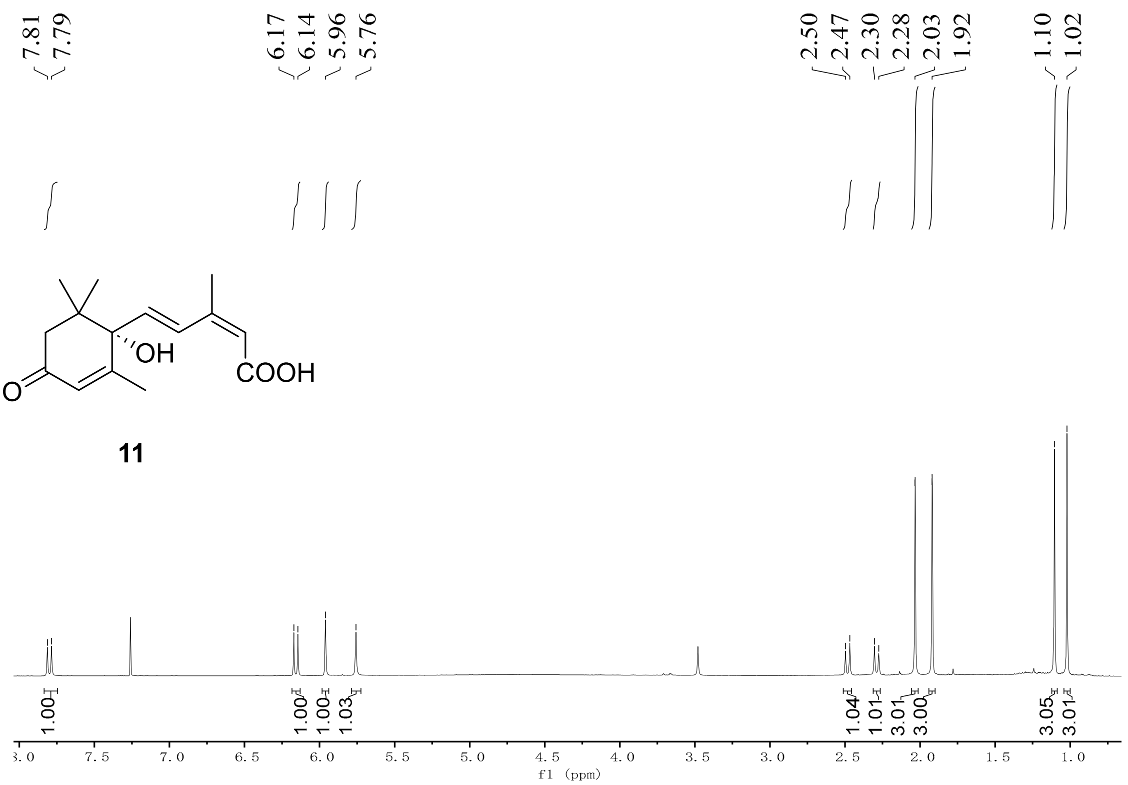

Figure S42. ^{1}H NMR (600 MHz) spectrum of compound **11** in $CDCl_3$

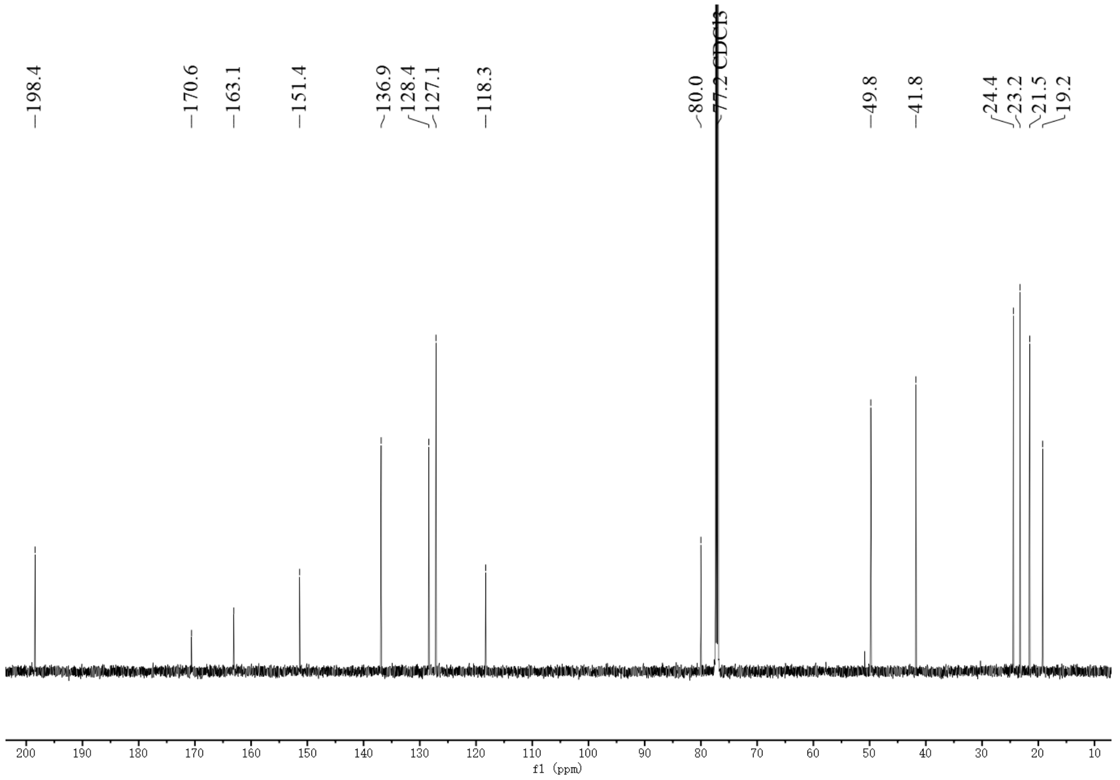

Figure S43. ^{13}C NMR (150 MHz) spectrum of compound **11** in $CDCl_3$

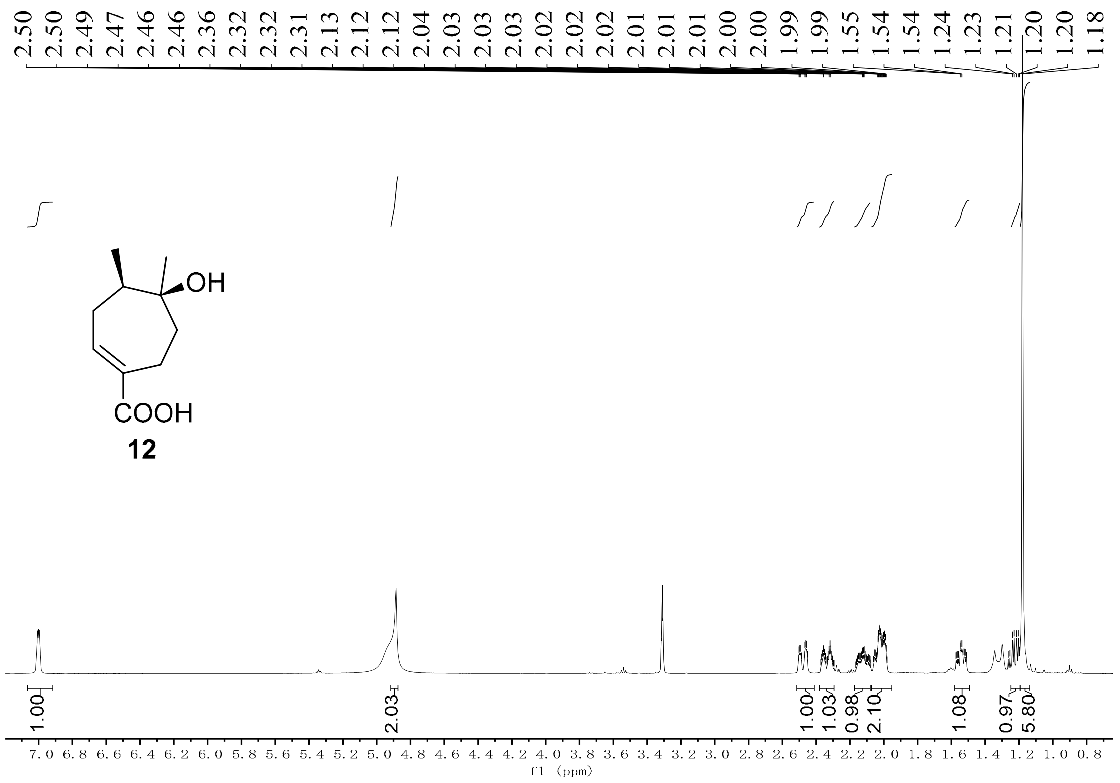

Figure S44. ^{1}H NMR (500 MHz) spectrum of compound **13** in CD_3OD

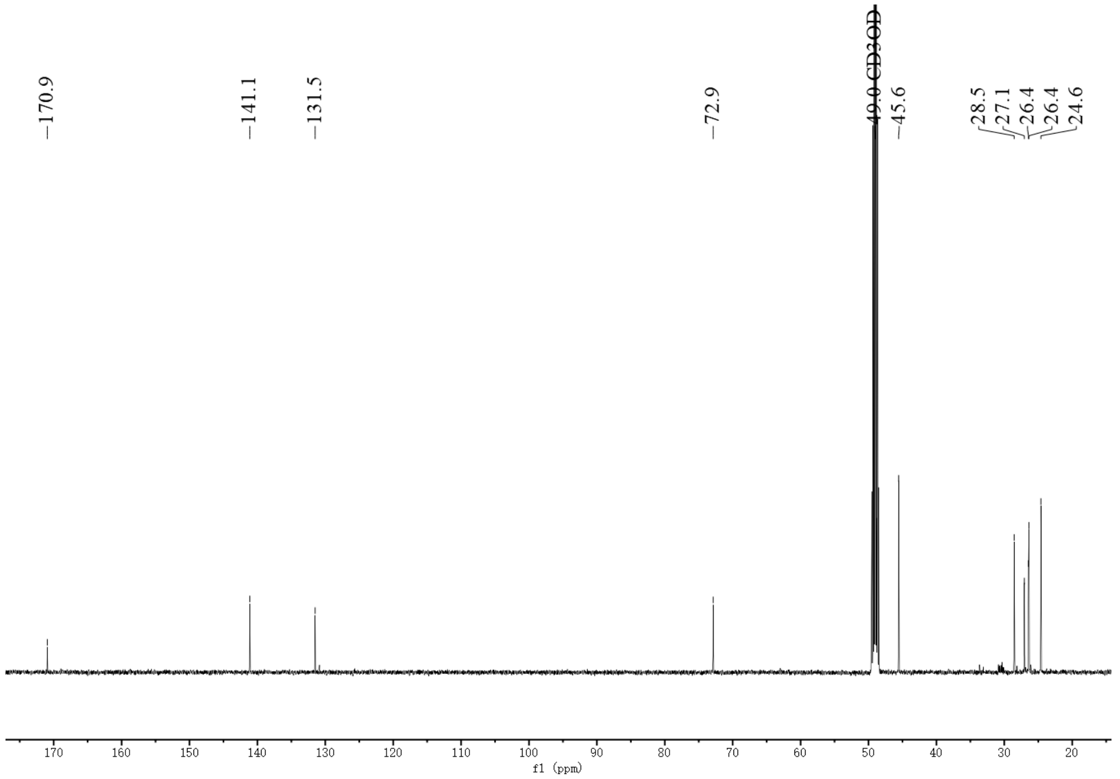

Figure S45. ^{13}C NMR (125 MHz) spectrum of compound **13** in CD_3OD

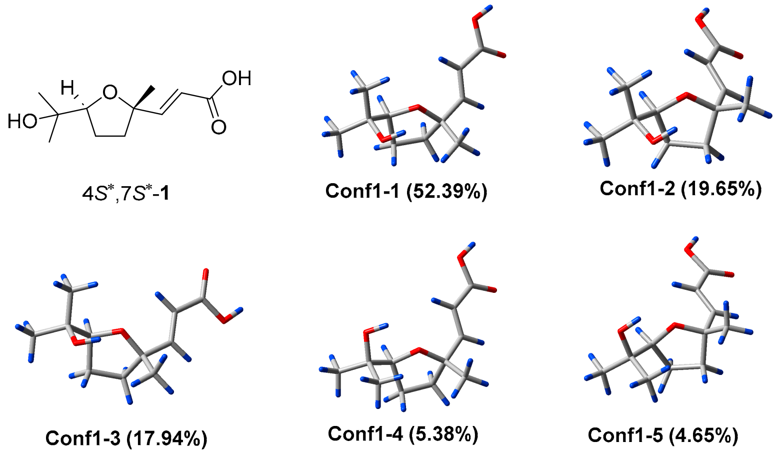

Figure S46. Optimized geometries of 5 dominant conformers of **1** at the B3LYP/6-31G(d) level of theory in acetonitrile (IEFPCM)

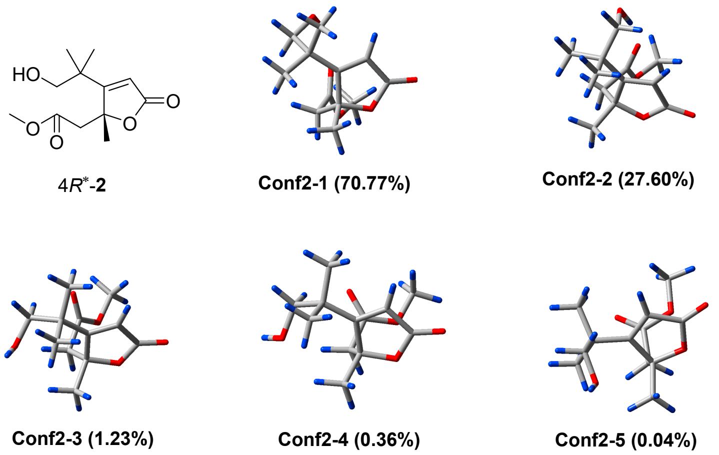

Figure S47. Optimized geometries of 5 dominant conformers of **2** at the B3LYP/6-31G(d) level of theory in acetonitrile (IEFPCM)

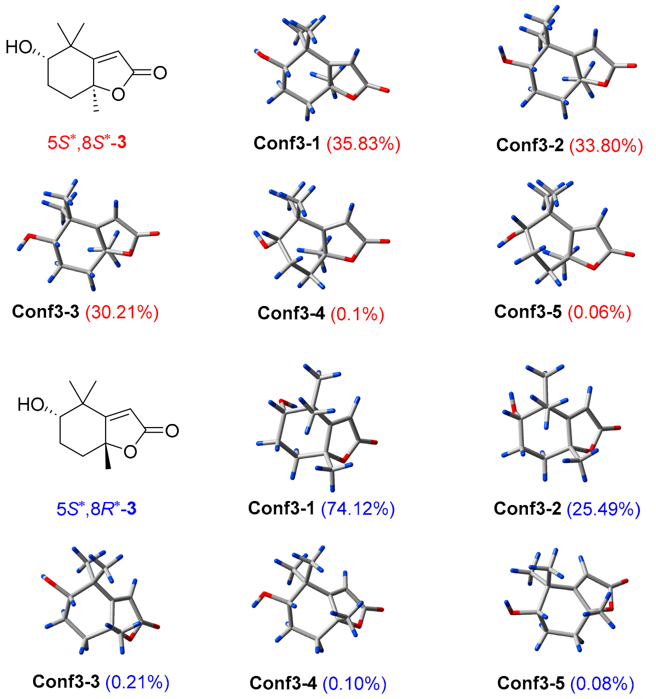

Figure S48. Stable conformers used in ^{13}C NMR calculation of **3**

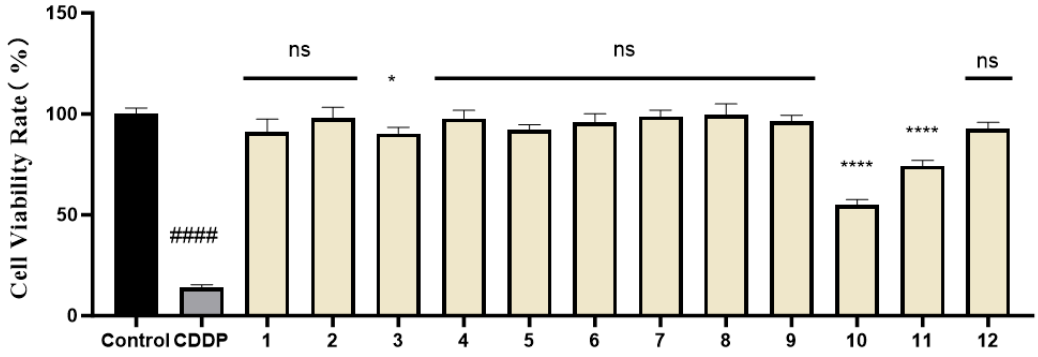

Figure S49. Effects of isolated compounds on the viability of A549 cells. A549 cells were incubated with compounds **1**–**12** or cisplatin (CDDP; positive control) at 20 μM for 72 h, and cell viability was determined by the MTT assay. Each concentration was tested in triplicate wells, and data are presented as the mean ± SD from at least three independent experiments. * $p < 0.05$, **** $p < 0.0001$, #### $p < 0.0001$ versus the control group; ns indicates no significant difference.

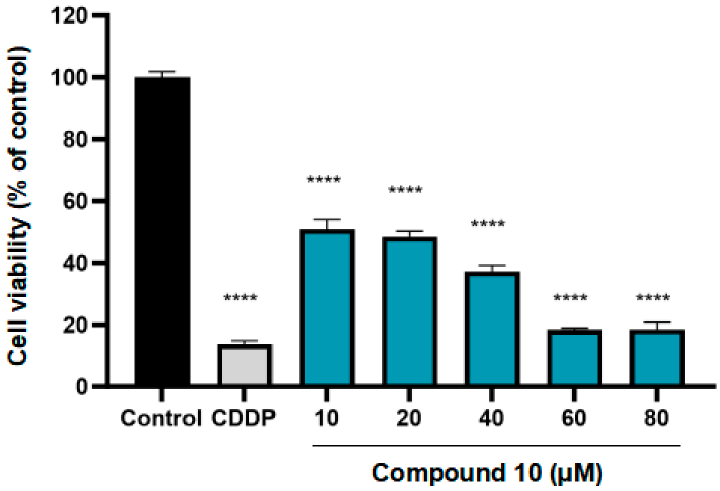

Figure S50. Concentration-dependent antiproliferative effect of compound **10** on A549 cells. A549 cells were exposed to compound **10** at concentrations of 10, 20, 40, 60, and 80 µM for 72 h, followed by assessment of cell viability using the MTT assay. CDDP served as the positive control. Data are presented as the mean ± SD. **** $p < 0.0001$ compared with the control group.
